# Understanding citizens’ attitudes within user-centered digital health ecosystems: A sequential mixed method methodology including a web-survey

**DOI:** 10.1177/20552076241255929

**Published:** 2024-05-20

**Authors:** Robin Huettemann, Benedict Sevov, Sven Meister, Leonard Fehring

**Affiliations:** 1 Faculty of Health, School of Medicine, Witten/Herdecke University, Witten, Germany; 2 Healthcare Informatics, Faculty of Health, School of Medicine, Witten/Herdecke University, Witten, Germany; 3 Department Healthcare, 28476 Fraunhofer Institute for Software and Systems Engineering ISST, Dortmund, Germany; 4 Gastroenterology, 60865 HELIOS University Hospital Wuppertal, University Witten/Herdecke, Wuppertal, Germany

**Keywords:** Digital health, health informatics, technology, apps, mixed methods, qualitative, quantitative

## Abstract

**Objective:**

Transitioning from digital health applications to digital health ecosystems, leveraging the advances in technologies and informatics, could be the next revolution in digital health. This includes offering centralized access to various health services and improving citizens’ well-being, delivery, clinical processes, and data management. However, a limited understanding of citizens may impede adaptation. Therefore, this study investigates citizens’ attitudes within digital health ecosystems, differentiated by their characteristics, to support health service-providers and governmental policymakers in establishing user-centered solutions.

**Methods:**

This study follows a three-step sequential mixed method methodology: (1) a literature review. (2) Qualitative thematic analyses based on semi-structured qualitative interviews. (3) Quantitative analyses based on a web-survey (descriptive statistics, one-way analysis of variances, Tukey-honestly, and Cohen's d tests).

**Results:**

N = 15 citizens were interviewed and n = 1289 responded to the web-survey, to our knowledge the largest survey on this topic. Citizens desire a more convenient management of health services and data (M = 5.2, SD = 1.59). Services with peer-to-peer interactions (M = 3.7, SD = 1.81) and lower involvement of health professionals (M = 3.8, SD = 1.75) are less demanded. Data protection is critical (M = 6.2, SD = 1.23). Public payers are mandated as orchestrators (M = 4.3, SD = 1.99), while private companies receive lower acceptance (M = 3.0, SD = 1.42).

**Conclusions:**

Health service-providers could follow a three-staged approach to establish digital health ecosystems: (1) Increasing the convenience for citizens by enabling online management of health services and data. (2) Extending the citizen–healthcare provider partnership through online interactions. (3) Fostering preventative behaviors and quicker recovery by personalizing health services and interactions. Governmental policymakers should integrate an electronic health record.

## Introduction

Advances in the use of the internet, technologies, and informatics in healthcare,^1^ including electronic Health (eHealth)^⁠^^2^ or mobile Health (mHealth),^3^ have been collectively summarized under the umbrella term digital health,^4^ which is defined by the World Health Organization as “*[…] development and use of digital technologies to improve health. Digital health expands the concept of eHealth to include digital consumers, with a wider range of smart-devices and connected equipment. It also encompasses other uses of digital technologies for health such as the Internet of things, artificial intelligence, big data and robotics*.”^5^ Even before the COVID-19 pandemic, there were 3.7 billion commercially available digital health applications (apps).^6^ The pandemic,^7^ along with the falling costs for associated supporting technologies and devices, such as wearables,^8^ accelerated the availability of digital health apps. However, digital health apps are rarely used in the long term. For example, only 4% of mental health app users have stated to continue to engage with these apps meaningfully after 15 days,^9^ likely driven by three limitations: First, digital health apps are often self-service oriented and centered around a “single-issue,”^10^ while citizens increasingly seeking integrated solutions that address all their health needs, including accessing health services and managing personal health data. Yet, they are required to engage with multiple digital health apps, leading to a reduction in perceived added value and may affecting adherence.^6,10–12^ Second, services that involve digital rather than professional–personal interactions often lack integration within a joint journey,^6,11,13^ leading to inconveniences.^8,14,15^ Third, the perspective has expanded beyond patient care, shifting from a focus on diagnosis and treatment to citizens’ overall well-being,^16^ respectively, from disease to health journeys, including preventative and payment-related services.^17,18^

Addressing these limitations by leveraging the internet, technologies, and informatics has given rise to digital health ecosystems, often considered the next revolution in improving citizens’ well-being, health service delivery, clinical practices, health system processes, and data management.^13,19^ This revolution comes with profound cultural, social, and behavioral as well as economic implications for citizens, health service-providers, and governmental policymakers.^20^

For this study, the definition of digital health ecosystems is based on a combination of multiple frameworks/definitions used by previous research^12,13,21–26^:

“Digital health ecosystems are citizen-facing online applications (apps) such as mobile apps and web interfaces, which integrate, complement, and facilitate access to various digital and offline (professional-personal) health services along the health journey. Typically, these services are provided by various health service-providers on the supply side (incl. public and private healthcare providers, public payers, private insurers, governmental institutions, technology companies, start-ups, pharmaceutical companies). These health service-providers share a common vision of improving citizens’ well-being while enabling the convenient delivery of health services and trusted interactions with citizens on the demand side. This is orchestrated by one health service-provider assuming the role of a leader, operating within the regulatory and infrastructure standards set by governmental policymakers.”

Digital health ecosystems promise benefits for all stakeholders: governmental policymakers may expect benefits from addressing broader health system challenges, primarily the rising health supply gap (e.g. due to a shortage of skilled labor) and health system expenditures.^27–29^ Some expect health service-providers that emerge as successful orchestrators to become among the largest companies in the world.^30^ As a first proof, PingAn, a private insurer, has successfully established a digital health ecosystem in China^31^ and has been ranked as the highest valued insurance brand since 2020.^32^ However, according to previous research, health service-providers primarily anticipate benefits regarding improved efficiencies in their clinical and health processes as well as health services delivery, including interactions with citizens.^7,13,27,33,34^ Relative to health service-providers, citizens could benefit even more,^20^ potentially in three aspects: Firstly, from increased convenience in interactions and access to health services, e.g. through shorter waiting times^⁠^^6,19,35^ or better health data management.^6^ Secondly, from enhanced partner-like relationships between patients and healthcare providers^24,36^ as patients become more empowered^26^ and engaged.^37,38^ Thirdly, from improved health and overall well-being, e.g. through enhanced awareness,^39^ preventative health warning accuracies,^24,40^ medical treatments,^41–43^ and access to care in emergencies.^44^

Despite potential benefits for all stakeholders, in most countries, a digital health ecosystem has yet not been broadly established.^31^ Given this absence, this study focuses on citizens’ attitudes within user-centered digital health ecosystems, recognizing the necessity to first establish a detailed understanding of the citizen (demand) perspective^10,12,14,21,30,45–48^ to support decision-makers at health service-providers and governmental policymakers prioritizing their actions to develop user-centered digital health ecosystems with lasting adaptation.

Thereby, the sample of this study focuses on Germany following two considerations: first, German citizens indicate an interest in using digital health ecosystem, with 40% reporting their use of digital health apps at least weekly.^49^ Second, while citizens’ expectations have been investigated in certain countries (e.g. Australia,^12^ Pakistan,^50^ Qatar^51^), the applicability of these findings to countries globally remains limited due to distinct health systems, suggesting the need for country-specific sampling.^50,52^ While acknowledging this, this study aims for findings that have relevance to countries internationally. Referring to Sheikh and Ghafar (2021), health system structures can be described using four indicators, each with different optional metrics for comparability: (1) governance (e.g. based on “availability, or scope of a national ‘Digital Health Strategy’”), (2) financing (e.g. based on mandatory versus supplementary health insurance, and public, employer, or private funded), (3) resources (e.g. based on “percentage share of GDP spent per capita on health expenditures,” or “ratio of physicians per 1000 population,” and (4) Service Organizations (e.g. based on “private versus public health service providers such as hospitals,” or “accessibility as a measure of service organization quality”).^53–56^ Across these indicators and their associated metrics, Germany can be considered average among countries, such as those in the OECD.^54,56^ Noteworthy, regarding “financing,” Germany with its distinctive dual health system integrates various health system structures used by countries globally into a single health system, encompassing both public payers and private insurers, as well as mandatory and supplementary insurance structures. Thus, citizens can choose between public payers and private insurers for their mandatory health insurance. Supplementary health insurance, which reimburses for offerings beyond the mandatory health insurance, is primarily offered by private insurers and can be chosen optionally by citizens.^28,57,58^

Hence, we are convinced that findings derived from a German study sample have relevance to countries internationally, independently from their health system.

Along the four indicators used to compare health system structures internationally, compared to Germany, countries such as Australia, Italy, Canada, Sweden, and the United Kingdom exhibit similar structures. Hence, the results of this study may be particularly relevant for these countries. For example, all have a “digital health strategy” and allocate a “percentage share of GDP spent per capita on health expenditures” within plus or minus three percentage points compared to Germany. Additionally, they align with OECD averages in terms of “service organization” indicators. Furthermore, each of these countries, like Germany, has a mandatory health insurance system in place. While for the mandatory health insurance, the primary source of financing for the majority of citizens is public sponsorship, supplementary health insurance is typically received through private insurers and privately funded. However, Canada (due to employer sponsorship) and Australia have a more equal split between public and private funding as the primary funding sources for mandatory health insurance.^54,56^

To the best of the authors’ knowledge, existing studies on German citizens exhibit limitations, focusing on narrow sub-groups (e.g. pregnant women^26^), specific aspects (e.g. data protection^59^), or disregarding potential shifts due to the Covid-19 pandemic (e.g. published prior 2020^60^).

Guided by the overarching objective of supporting governmental policymakers and decision-makers at health service-providers globally in establishing user-centered digital health ecosystems, this study targets the following research question:

“What are the attitudes of citizens within digital health ecosystems along the health journey, considering 1) added values, 2) services and interactions, 3) digital characteristics, and 4) health service-providers, and how do these attitudes vary based on citizens’ personal characteristics?”

## Methods

### Overview: three-step sequential mixed method methodology

This study follows a three-step sequential mixed method methodology. In compliance with the “Mixed Methods Article Reporting Standards” (MMARS), the methodology and results of each of the three steps are reported separately in the following.^61^

The starting point was a literature review, which itself did not serve as a standalone data source for this research study but informed the following sequential qualitative and quantitative primary data collection and analysis approaches. From the literature review, potentially relevant dimensions (referred to as a priori codes) along the research questions were derived. Secondly, based on the literature review, an interview guide containing open-ended questions was designed to collect primary data via semi-structured qualitative interviews (referred to as interviews). These interviews aimed to generate exploratory findings, analyzed both qualitatively and semi-quantitatively.^62^ These analyses followed a thematic approach by assigning further codes to relevant results. Subsequently and thirdly, the combined coding resulting from the literature review and interviews served as input for the answer items of the web-survey questionnaire. The collected primary data from the web-survey questionnaire, which contained answer items derived from the combined results of both the literature review and interviews to ensure all relevant answer items were considered given its closed format, aimed to generalize the findings through quantitative analysis.

The “Ethics Committee of the Witten/Herdecke University” (No. S-213/2022) raised no objection regarding ethical concerns.

### Step 1: literature review as secondary data source

**Figure 1.**
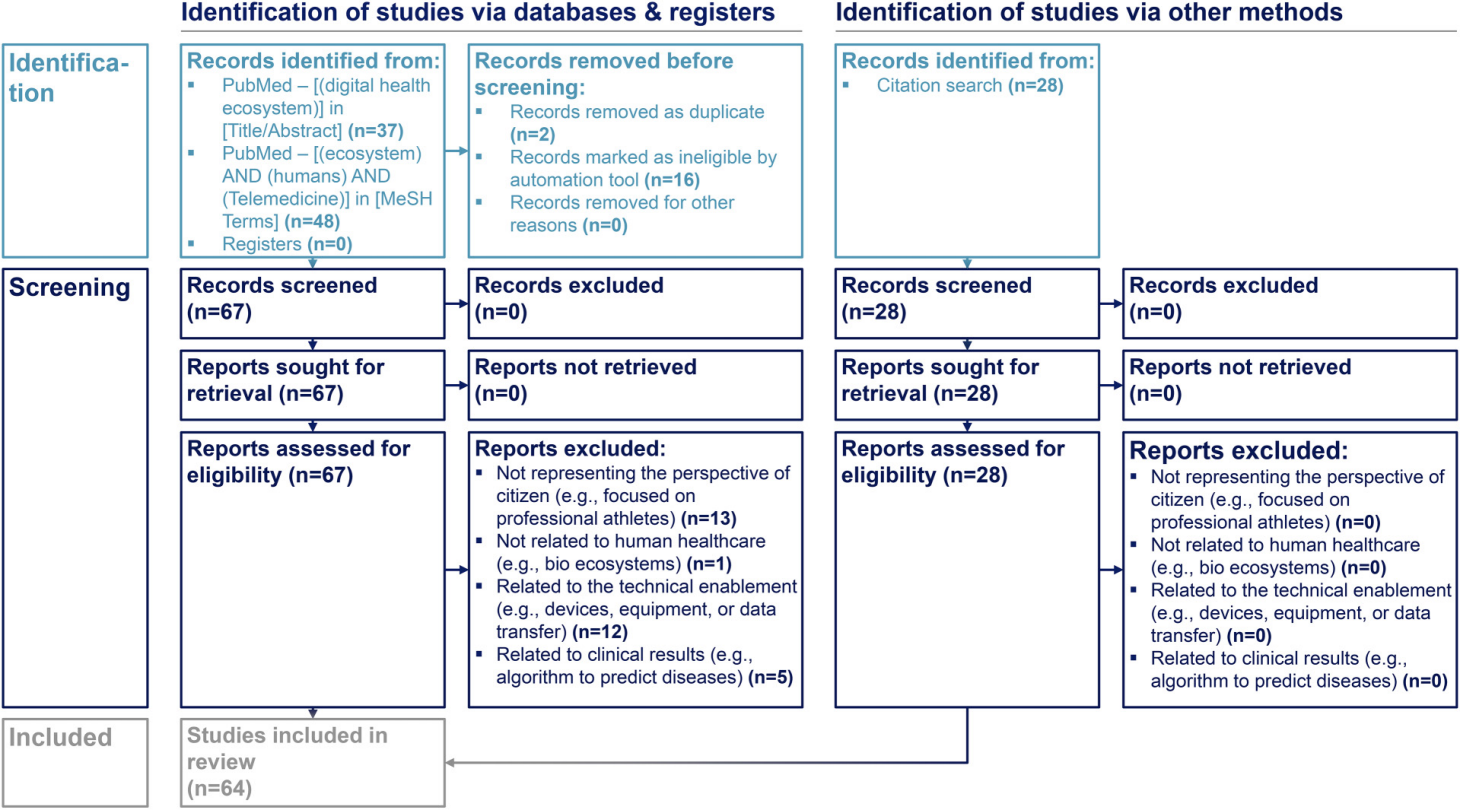
Included studies form the literature review. Identification and screening process to include relevant literature guided by the PRISMA-approach for literature scoping reviews.^63–65^

The literature review was guided by the steps recommended by the PRISMA-approach for scoping reviews (RISMA-ScR).^63–65^ Figure 1 presents the identification and screening process to include relevant studies.

Inspired by Alvandi et al.,^12^ the search strategy included the following search terms:

“ecosystem [MeSH Terms]) AND (humans [MeSH Terms]) AND (Telemedicine [MeSH Terms])”

“(digital health ecosystem [Title/Abstract])”

*“*PubMed” was used as search engine. Identification criteria included “English language” and “human species” as filters set in the database automation tool. Duplicates were removed. The title and abstract of the records were screened by two co-authors and excluded if the records were incorrectly classified as eligible by the database's automation filter tool. The remaining records were assessed in their full length for eligibility and excluded if they focused on non-representative citizen groups (e.g. professional athletes), non-human ecosystems (e.g. bio ecosystems), technical aspects (e.g. on hardware), or clinical research (e.g. performance of algorithm to predict diseases). In addition, citation search was applied based on the included studies identified through the “PudMed” search engine (see Figure 1).

Included studies were synthesized by deriving a priori codes along the research questions through two co-authors. One co-author allocated the initial a priori coding along the research question, merged a priori codes if they were not distinct, or renamed them. A priori codes were assigned for relevant results, respectively, findings mentioned by the included studies. A second co-author reviewed the a priori codes. They were either confirmed or discussed between the two co-authors until both were confident with the a priori coding as the basis for further refinement, as part of the sequential mixed method approach. The a priori codes derived from the literature review results served as initial coding for the thematic analysis of the interviews. Sequentially, these a priori codes were detailed and supplemented through the interview results, resulting in the combined final coding. Ultimately, this combined coding served as input for the answer items of the web-survey questionnaire, given its closed format.

### Step 2: semi-structured qualitative interviews as primary data source analyzed using thematic analyses

The interview guide is structured along the research question and includes six sections: (1) general information and digital health ecosystem definition, (2) added value (e.g. “quick access to health needs,” etc.),^13^ (3) services and interactions (e.g. “online health history/data exchange,” etc.),^12^ (4) digital characteristics—related to citizens’ general attitudes toward the requirements and conditions for adapting to digital health ecosystems—(e.g. “data security,” etc.),^10^ (5) health service-providers, and (6) personal characteristics as predictor variables (gender and age) (Supplementary Material 1 presents the complete interview guide). All participants were informed in advance in writing about privacy considerations and the scope of the research.

The interview data were analyzed using a thematic analysis, following the approach described by Braun and Clarke, to explore qualitative reasoning and semi-quantitative insights.^62^ In this context, all interview data were translated into factually anonymized transcripts, ensuring privacy interests.^66^ Interview transcripts were coded by two co-authors, who first familiarized themselves with the transcripts, followed by three rounds of coding using MAXQDA (Version 2022.4). First, statements, if mentioned as relevant by the participants, were either assigned to one of the a priori codes for detailing or supplemented as distinct new codes. Second, codes were revised (detailed, renamed, or merged). Third, a second co-author reviewed the coding. Codes were either confirmed or discussed until both were confident that the coding represented an adequate representation of the interview data (Supplementary Material 2 presents further details on the methodology, and Supplementary Material 3 includes the code names and descriptions).

For the semi-quantitative analysis, the frequency per code per participant was limited to one, to avoid skewed results if a participant referred disproportionately often to one code only. Interviewing continued until the two co-authors, serving as coders, agreed that content saturation was evident, expressed through the repetition of codes.^62^

The interview guide, design, and analytical approach were tested through three test interviews, which were not included in the final data set.

The approach was guided by and details, including participant recruitment and selection, are reported according to the “Consolidated criteria for reporting qualitative research (COREQ): a 32-item checklist for interviews and focus groups”^67^ (see Supplementary Material 2).

### Step 3: web-survey as primary data source analyzed using one-way analysis of variances, Tukey-honestly significant difference, and Cohens’ d tests

Overarchingly, given that a digital health ecosystem does not yet exist in Germany at scale,^31^ within the quantitative part of the mixed method approach, it was the objective to approximate “real citizens’ behaviors” using a “health preference research” (HPR) methodological approach as substitute. Several quantitative HPR methodological approaches were considered, which can be distinguished twofold, based on what they are measuring. While approaches such as pairwise comparisons, conjoint analysis, or discrete choice experiments measure stated preferences, rating approaches are especially suitable to measure attitudes. Thus, it is an “attitudes versus stated preferences” question. While both HPR methodological approaches have different dis-/ advantages, choosing the most fitting HPR methodological approach is a trade-off specific to the research context.^68^

Key considerations for this trade-off include the following. The results of both HPR methodological approaches appear to be similar for the attributes rated in the top and bottom groups, with differences only in the order of attributes within the top, respectively, bottom groups themselves.^68^ Rating approaches have proven to provide better results for questions with foundational compared to questions with difficulty, frequency, or severity characteristics.^69^ The main difference between the two HPR methodological approaches is the resource constraint format leveraged by stated preference measuring approaches. The advantage of a resource constraint format is the decomposition, which requires respondents to make trade-offs between attributes, better approximating a “real preference.” This might also better approximate “real behaviors” and provide a better understanding of the underlying decision process. The disadvantage is that respondents often perceive resource constraint formats as “more difficult to answer,” leading them to use cognitive heuristics to simplify decision-making, potentially resulting in inconsistent responses.^68,69^

Linking this trade-off to this study's research context, the following five considerations determined the use of an attitude measuring Likert scale-based rating HPR methodological approach. The first consideration links to the rather “foundational” characteristic of this research question, as citizens’ attitudes regarding digital health ecosystems have not yet been explored at scale in Germany. Therefore, second, it was not desired to force respondents to make trade-offs between attributes, as it could lead them to downgrade ones that are important to them. Consequently, respondents were allowed to potentially rate all attributes equally important (as a commodity), and to rate all attributes independently, regardless of others. Third, the perceived “more comfortable to answer” format allowed for the recruitment of more respondents and the integration of more attributes, particularly when conducted as a web-based survey, facilitating the creation of a larger data sample with a higher likelihood of consistent answers per respondent. Following this reasoning, fourth, it may be easier to repeat the study in other countries internationally while generating results with a higher comparability.^68,69^ Five, the use of rating questions based on Likert scales measuring attitudes is common in international (digital) health-related patient, respectively, citizen research, and was exemplarily used by Weik et al. (2024),^70^ Zanaboni and Fagerlund (2020),^71^ Cao et al. (2023),^72^ Zhu et al. (2022),^73^ Mayer et al. (2022),^74^ or Lee et al. (2022),^75^ among others.

The web-survey questionnaire is structured along the same six sections as the interview guide. The answer items of the closed questions are based on the identified (a priori) codes, originating either from the literature review or the interviews. Each answer item reflects one dependent variable, which is to be rated using a one-to-seven-point Likert scale. An additional eighth “no answer preferred” option was available. The questions about services and interactions are structured along the health journey,^17,18^ including four steps: prevention, diagnosis, treatment, and (health insurance) payment.^17^ Section six contains a comprehensive set of 12 personal characteristics as predictor variables (each based on question), respectively, constructs (each based on more than one question), allocated along four categories: (1) “Demographics” including “age,” “gender,” “education level” and “employment relationship” (one question for each); 2) “health status” including “number of healthcare provider visits (last 12 months)”, “health feeling,” “health interest” (questions and scales of the latter two are applied as introduced by Nunes, Coyle, and Bardram^76^), and “permanent/chronic illness” (one question for each); (3) “Vienna Patient Satisfaction Inventory” (VPSI) including the constructs “access to personal treatments” (five questions), “competence of treatment staff” (two questions), and “effectiveness of personal treatments” (three questions)^77^; (4) “Affinity for Technology Interaction” (ATI) construct (nine questions).^78^ The questions and scales used to evaluate the “VPSI”^77^ and “ATI”^78^ constructs were introduced by prior research and applied accordingly. The selected personal characteristic categories and underlying predictor variables (constructs) closely align with those used by Russel et al. Consequently, “personality traits” were not used as predictor variables, as Russel et al. have shown that these have limited predictive power for the intention to use digital health services.^79^ The questionnaire includes one control question to check the attention of the respondents (Supplementary Material 3 presents the complete questionnaire).

**Figure 2.**
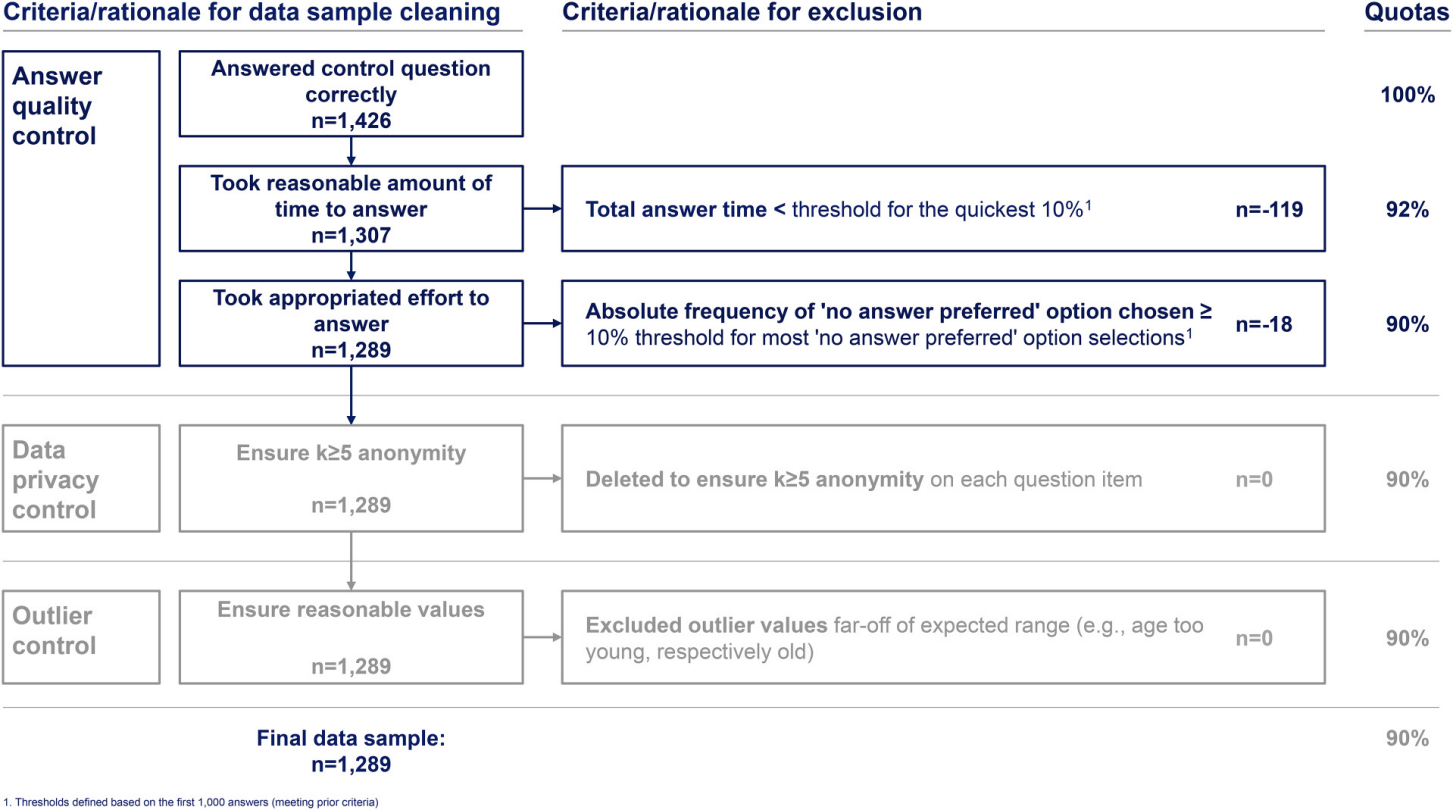
Web-survey data sample cleaning criteria/rationale. Only responses from respondents who completed the entire web-survey and correctly answered the control question were considered. The data were filtered to exclude respondents who did not take a reasonable amount of time to answer the questions. “Reasonable time” was defined as taking more time to answer than the quickest 10% of respondents. This threshold in absolute terms was determined after reviewing the first 1000 complete responses with correct control question answers. Respondents who answered more quickly than this absolute threshold were excluded. The established threshold was then applied to all subsequent respondents for exclusion, resulting in the exclusion of 119 responses. Applying a similar rationale, 10% of respondents who most frequently chose the “no answer preferred” option were excluded based on an absolute threshold defined using the first 1000 complete responses with correct control question answers. The established threshold was then applied to all subsequent responses for exclusion, resulting in the exclusion of 18 further responses. Noteworthy, the respondents who answered the quickest significantly overlapped with those who most frequently chose the “no answer preferred” option. In the further cleaning process, zero responses were excluded to ensure a minimum anonymity level of k ≥ 5 and no outliers were identified.

Ten citizens tested the web-survey. Their responses were not included in the data set. Figure 2 presents the web-survey data cleaning criteria and rationale. First, the data from the web-survey were cleaned, ensuring answer quality and privacy, while also removing outliers. To ensure data privacy interests, the results were analyzed with k ≥ 5 anonymity, meaning all answer items with n < 5 responses were deleted and excluded from the analyses. After the cleaning, the final data were tested for statistical usefulness through a multicollinearity test (Pearson correlation) to ensure that the predictor variables do not express the same relationship and internal consistency tests (Cronbach's alpha) for the used “VPSI” and “ATI” constructs to ensure that the underlying questions are a good presentation for the respective predictor variable.

The web-survey data was quantitatively analyzed using descriptive statistics, including calculating arithmetic means (M) and standard deviations (SD). The further analysis followed three steps to identify significant differences between citizen groups, defined by different attributes within a certain personal characteristic, respectively, predictor variable (e.g. between men and women, or age groups): first, one-way analysis of variances (ANOVA) tests were conducted for each answer item per predictor variable (construct) to determine significant differences between groups. If significance was observed, Tukey-honestly significant difference (HSD) tests followed to identify the specific two groups within a predictor variable (construct) that showed the most significant differences in m ratings (M-delta). M-delta is defined as the absolute difference between the M for an answer item of one citizen group within a certain predictor variable (construct) and the M of another citizen group within the same predictor variable (construct). Third, the strength of the effects between the two identified groups was assessed using the Cohen's d (d) test, based on a pooled standard deviation.^80^ The significance level was set at a *p*-value (*p*) ≤ .05.^81,82^ Effects with a significance level at *p* ≤ .001 were termed highly significant.

For the analysis, selective predictor variables (constructs) were grouped (e.g. “age,” or “number of healthcare provider visits (last 12 months)”, etc.) (see Supplementary Material 3). IBM SPSS (Version 29) was used for performing the statistical analyses.

The approach was guided by and details are reported according to the “Checklist for Reporting Results of Internet E-Surveys (CHERRIES)” including 30 items^83^ (Supplementary Material 4 presents further details).

## Results

### Step 1: literature review including n = 64 studies

The included studies refer to the search conducted on 24 February 2023. A sample size (n) of 36 studies was included. In addition, 28 studies were added following the citation search (see Figure 1).

Based on the included studies, 39 a priori codes have been identified and added as answer items to the web-survey questionnaire (see Supplementary Material 3).

### Step 2: semi-structured qualitative interviews including n = 15 participants

A total of n = 15 participants were interviewed between 20 March and 14 April 2023, evenly distributed across age and gender groups: 2 females, 3 males <30 years; 3 females, 2 males ≥30 < 60 years; 3 females, 2 males ≥60 years (see Supplementary Material 5 presents the personal characteristics of the participants). Content saturation was reached with the fifth interview in each age group.

Sixteen additional codes were identified, supplementing the 39 a priori codes from the literature review. Thus, the web-survey questionnaire in total included 55 dependent variables, respectively, answer items (see Supplementary Material 3).

Among the 16 supplemented codes, the top three refer to increasing convenience through the online management of health needs and access to interact with health service-providers (e.g. payers, insurers, healthcare providers): first, “communication portal with payers and insurers” (93%).

“Participant 3 ‘[…] instant messaging in a chat-like format, which enables exchanging short messages’.”

Second, “online health history/data exchange” (87%).

“Participant 4 ‘I also want to check-in and provide all necessary information, like personal information, health history, e.g., allergies and insurance card digitally, rather than manually […]’.”

Third, “chat boots” (for the communication with payers, insurers, or healthcare providers) (80%), sometimes referred to in a negative notion.

“Participant 7 ‘But the chat function should be based on a human, I do not really want to chat with a KI or similar’.”

Among the 39 a priori codes, 32 (82%) were mentioned by participants. Consistently and almost exclusively, a priori codes associated with increasing convenience through the online management and access of health services and data emerged with the highest frequency. Thus, the top mentioned a priori codes along the research question were Perceiving an added value from “central coordination of health needs” (87%), demanding services and interactions like “central health data storage” (73%), “video consultation” (73%), and “personalized training offerings” (73%), appreciating digital characteristics like “quick access to relevant personal offerings” (80%), while mandating “governmental institutions” (93%) as orchestrator.

### Step 3: web-survey including n = 1289 respondents

**Figure 3.**
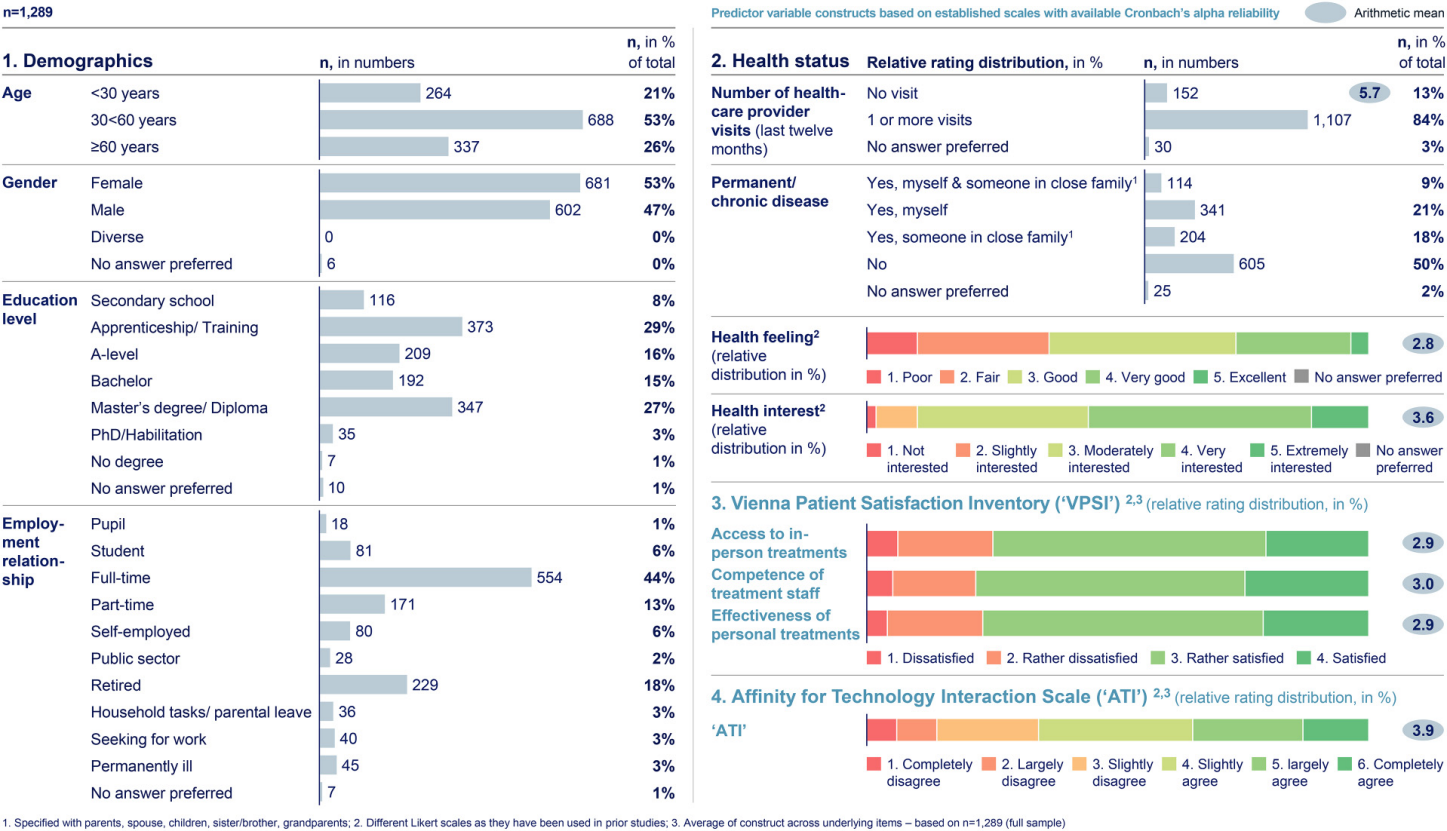
Personal characteristics of web-survey respondents by predictor variables (constructs). Based on primary data collected via a web-survey including n = 1289 German citizens. The final data sample shows diverse personal characteristics across the predictor variables (constructs). Respondents are evenly split across “age” and “gender” groups, with 45% having received academic education and 65% being either full-time-, part-time-, self-, or public sector-employed; 13% did not visit a healthcare provider within the last 12 months and 48% have some form of exposure to a permanent/chronic disease. On a one-to-five-point Likert scale, citizens rate their “health feeling” at M = 2.8 and their “health interest” at M = 3.6. On average, citizens express being “rather satisfied” with their personal healthcare provider experience and rate their “ATI” level with M = 3.9 on a one-to-six-point Likert scale.

The web-survey was accessible between 22 May and 30 June 2023. After the data sample cleaning process n = 1289 full responses were considered in the analysis (see Figure 2). To our knowledge this is the largest survey of citizens conducted on this topic in Germany. The data sample shows no multicollinearity between dependent variables on a Pearson Correlation < .7. The data indicate internal consistency for the used established constructs, with Cronbach's alphas ranging between .801 and .923 for the three constructs based on the “VPSI” and .902 for the “ATI” construct.^84^ These values are slightly higher than those originally reported.^77,78^ The M = 3.9 and SD = 1.3 for the “ATI” construct in this study are marginally higher than originally measured (M = 3.6, SD = 1.08).^78^ The web-survey respondents have diverse personal characteristics along the predictor variables (constructs) as presented in Figure 3.

In the following, the main results along the four research questions are outlined as well as illustrated using Tables 1 and 2, which show the univariate descriptive results of the web-survey as a rating distribution for all 55 dependent variables. Additionally, Tables 3, 4, and 5 show exemplarily one-way ANOVA and Tukey-HSD test results.

**Table 1.**
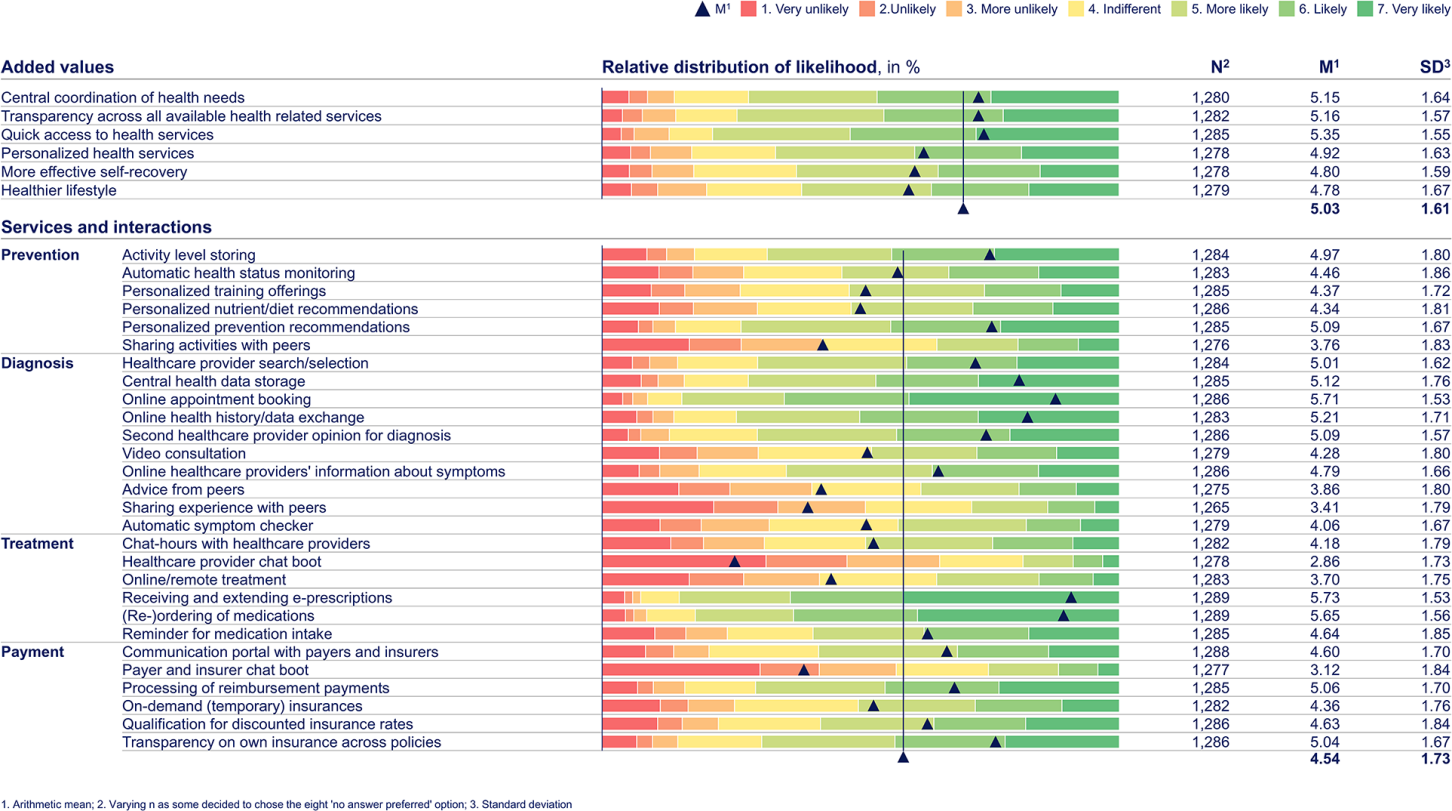
Web-survey rating distribution results by dependent variables for “added values” and “services and interactions.” Based on primary data collected via a web-survey including n = 1289 German citizens, using a one-to-seven-point Likert scale, with one being “very unlikely” and seven “very likely.” The web-survey included closed-ended questions based on a literature review and primary data from semi-structured qualitative interviews. In terms of added values, the top three ratings are associated with achieving more convenience through the online management and access of health services and data. Consistently, this perception among citizens also holds true with respect to the services and interactions. The top two services and interactions for each health journey step are as follows: “Personalized prevention recommendations” (M = 5.09, SD = 1.67), “activity level storing” (M = 4.97, SD = 1.80), “online appointment booking” (M = 5.71, SD = 1.53), “online health history/data exchange” (M = 5.21, SD = 1.71), “receiving and extending e-prescriptions” (M = 5.73, SD = 1.53), “(re-) ordering of medications” (M = 5.63, SD = 1.56), “transparency on own insurances across policies” (M = 5.04, SD = 1.67), and “processing of reimbursement payments” (M = 5.06, SD = 1.70). More innovative added values and services, related to online interactions with healthcare providers and personalized health, consistently show above-average standard deviations along the health journey, indicating their relevance to certain citizen groups, e.g. “personalized health services” (M = 4.92, SD = 1.63), “personalized nutrient/diet recommendations” (M = 4.34, SD = 1.81), “video consultation” (M = 4.28, SD = 1.80), “chat-hours with healthcare providers” (M = 4.18, SD = 1.79), “qualification for discounted insurance rates” (M = 1.84, SD = 1.84). Services linked to interactions with peers receive ratings below the M (M = 4,54, SD = 1.73), such as “sharing activities with peers” (M = 3.76, SD = 1.83) or “advice from peers” (M = 3.86, SD = 1.80). Services associated with reducing the interaction with healthcare providers receive below-average ratings, like “healthcare provider chat boot” (M = 2.86, SD = 1.73) or “automatic symptom checker” (M = 4.06, SD = 1.67).

**Table 2.**
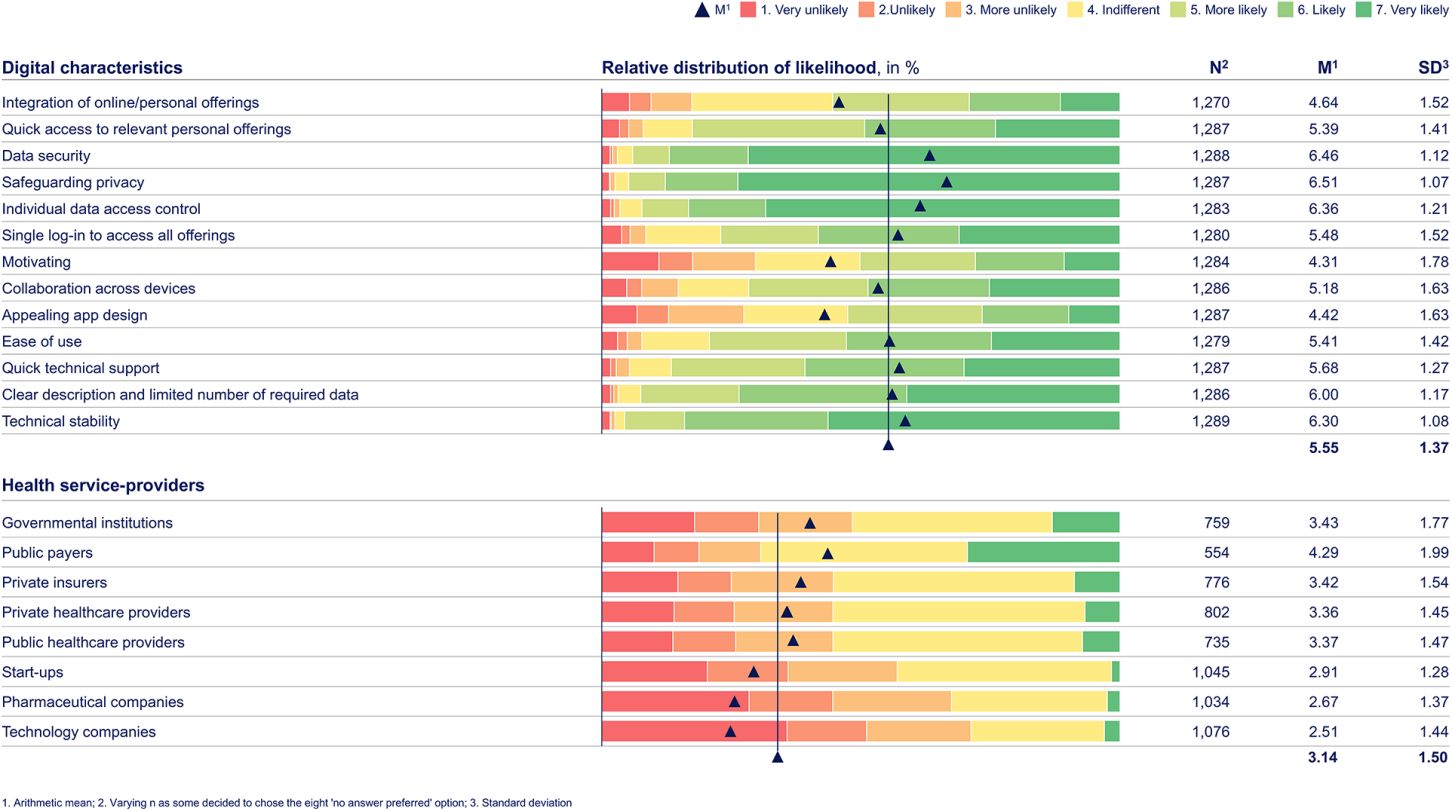
Web-survey rating distribution results by dependent variables for “digital characteristics” and “health service-providers.” Based on primary data collected via a web-survey including n = 1289 German citizens, using a one-to-seven-point Likert scale, with one being “very unlikely” and seven “very likely.” The web-survey included closed-ended questions based on a literature review and primary data from semi-structured qualitative interviews. Regarding the research question on digital characteristics, the three digital characteristics associated with data protection are rated above the m (M = 5.55, SD = 1.37): “Safeguarding privacy” (M = 6.51, SD = 1.07), “data security” (M = 6.46, SD = 1.12), and “individual data access control” (M = 6.36, SD = 1.21). Also, technical reliability-associated digital characteristics receive above-average ratings, including “technical stability” (M = 6.30, SD = 1.08) and “quick technical support” (M = 5.68, SD = 1.27). In terms of health service-providers, citizens mandate governmental and public institutions, as they were rated above the m (M = 3.14, SD = 1.50), like “public payers” (M = 4.29, SD = 1.99) and “governmental institutions” (M = 3.43, SD = 1.77). Others receive the lowest arithmetic means and standard deviations, indicating consistent rejection of these players as orchestrators.

**Table 3.**
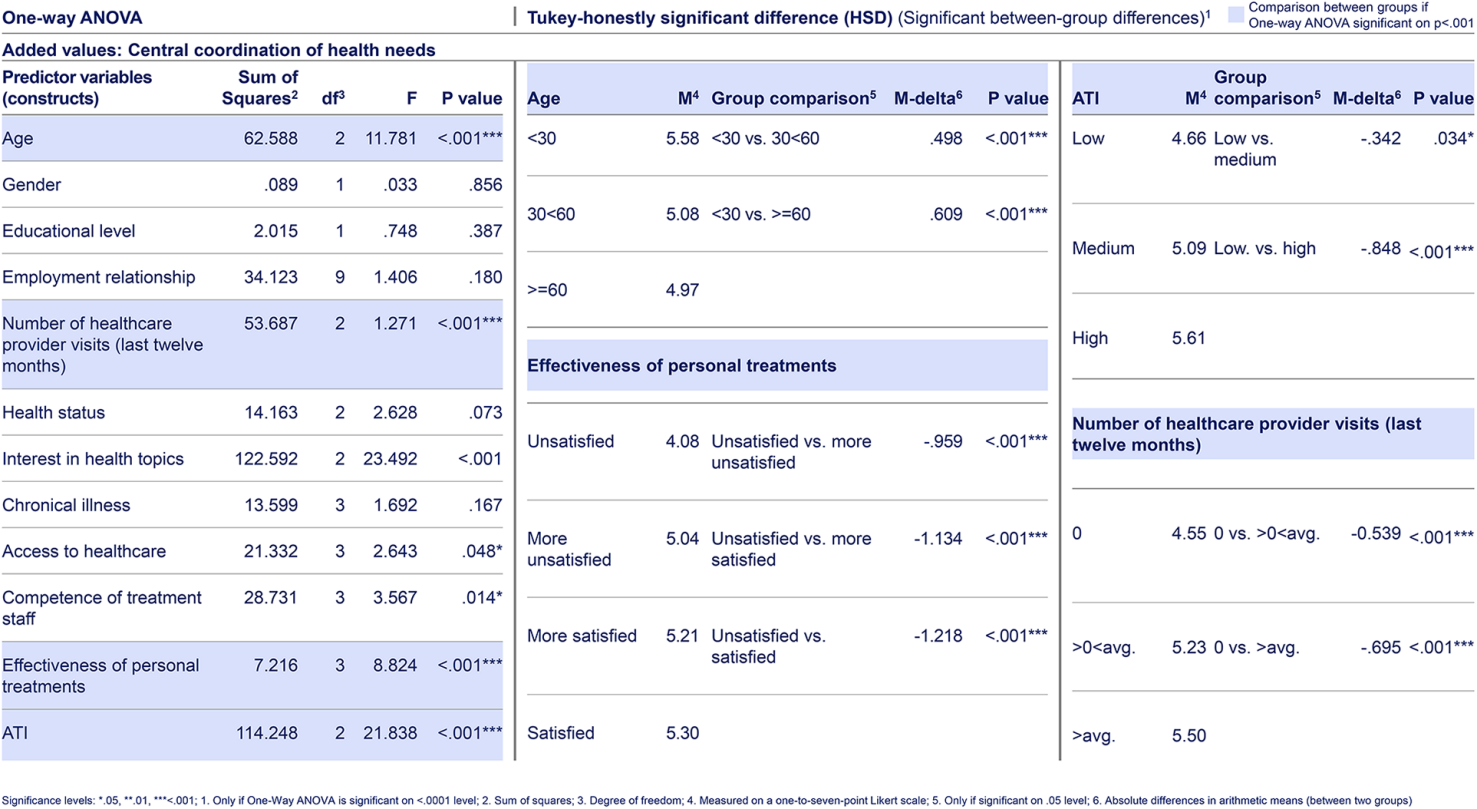
Selected web-survey one-way ANOVA and Tukey-HSD test result of citizens’ for “added values.” Based on primary data collected via a web-survey including n = 1289 German citizens, using a one-to-seven-point Likert scale, with one being “very unlikely” and seven “very likely.” The web-survey included closed-ended questions based on a literature review and primary data from semi-structured qualitative interviews. For each predictor variable (construct) per dependent variable, respectively answer item, a Tukey-HSD test was conducted, if the one-way ANOVA test indicated a highly significant difference between citizen groups in that predictor variable (construct). Overarchingly, the data consistently show that citizens with the highest “ATI” level, rate all aspects along the research questions higher, indicating to be more likely to use digital health ecosystems. In terms of the added value “central coordination of health needs”, the citizen groups who are the most versus the least satisfied with the “effectiveness of personal treatments” (*p* < .001, M-delta = 1.218, d = .64) and those with above-average versus without “number of healthcare provider visits (last 12 months)” (*p* < .001, M-delta = .695, d = .42) show the highest differences in m ratings.

**Table 4.**
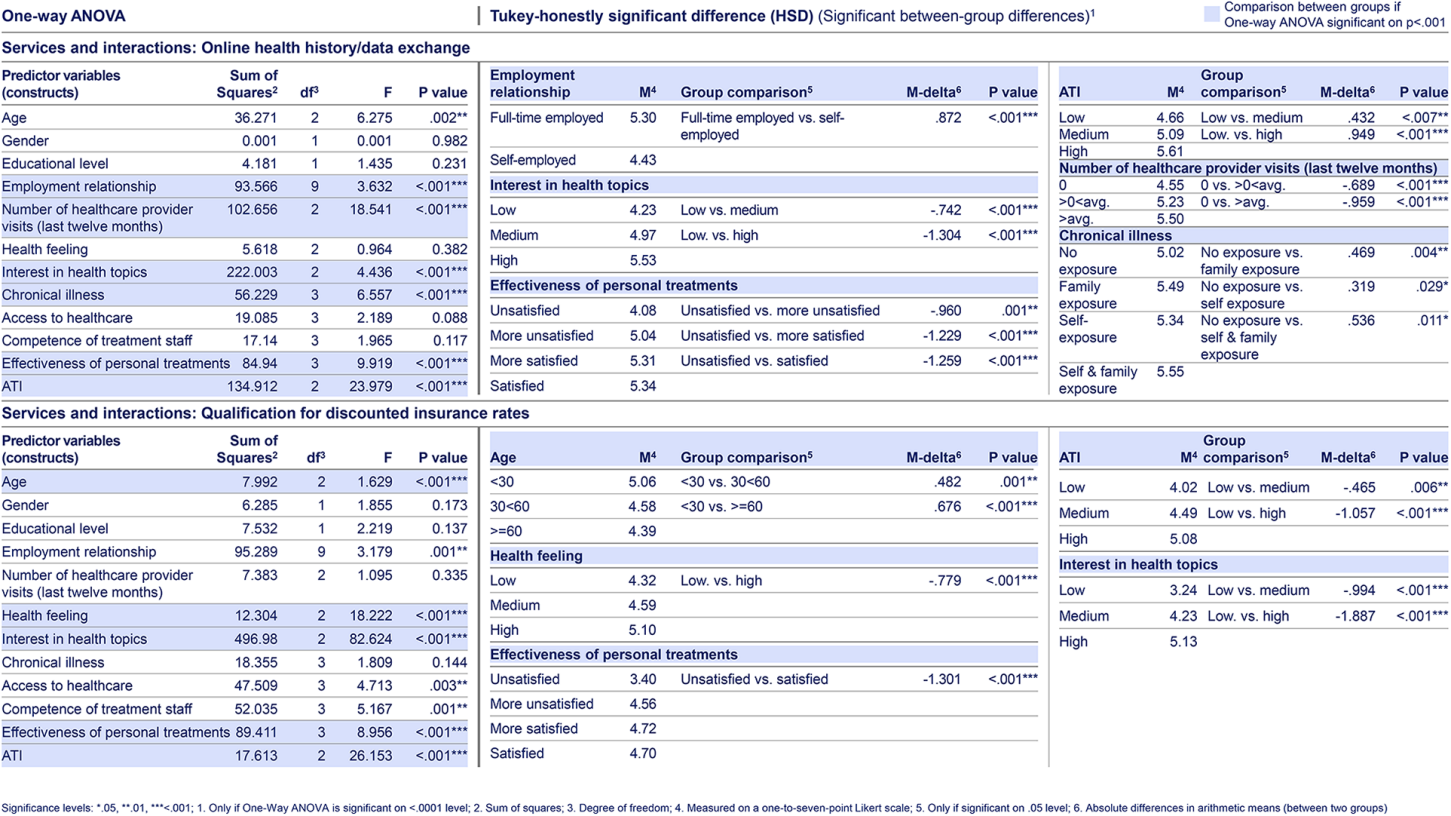
Selected web-survey one-way ANOVA and Tukey-HSD test results of citizens’ for “services and interactions.” Based on primary data collected via a web-survey including n = 1289 German citizens, using a one-to-seven-point Likert scale, with one being “very unlikely” and seven “very likely.” The web-survey included closed-ended questions based on a literature review and primary data from semi-structured qualitative interviews. For each predictor variable (construct) per dependent variable, respectively answer item, a Tukey-HSD test was conducted, if the one-way ANOVA test indicated a highly significant difference between citizen groups in that predictor variable (construct). The citizen groups who are the most versus the least satisfied with the “effectiveness of personal treatments” (*p* < .001, M-delta = 1.259, d = .65) and those with above-average versus without “number of healthcare provider visits (last 12 months)” (*p* < .001, M-delta = .959, d = .59) show the highest differences in M ratings regarding the services and interactions “online health history/data exchange.” The latter is also highly significantly more likely being used by the citizen group with a high versus low “interest in health” (*p* < .001, M-delta = 1.304, d = .79). The same citizen group (*p* < .001, M-delta = 1.887, d = 1.07) and citizen group with a high versus low “health feeling” (*p* < .001, M-delta = .779, d = .42) are highly significantly more likely to appreciate the “qualification for discounted insurance rates.”

**Table 5.**
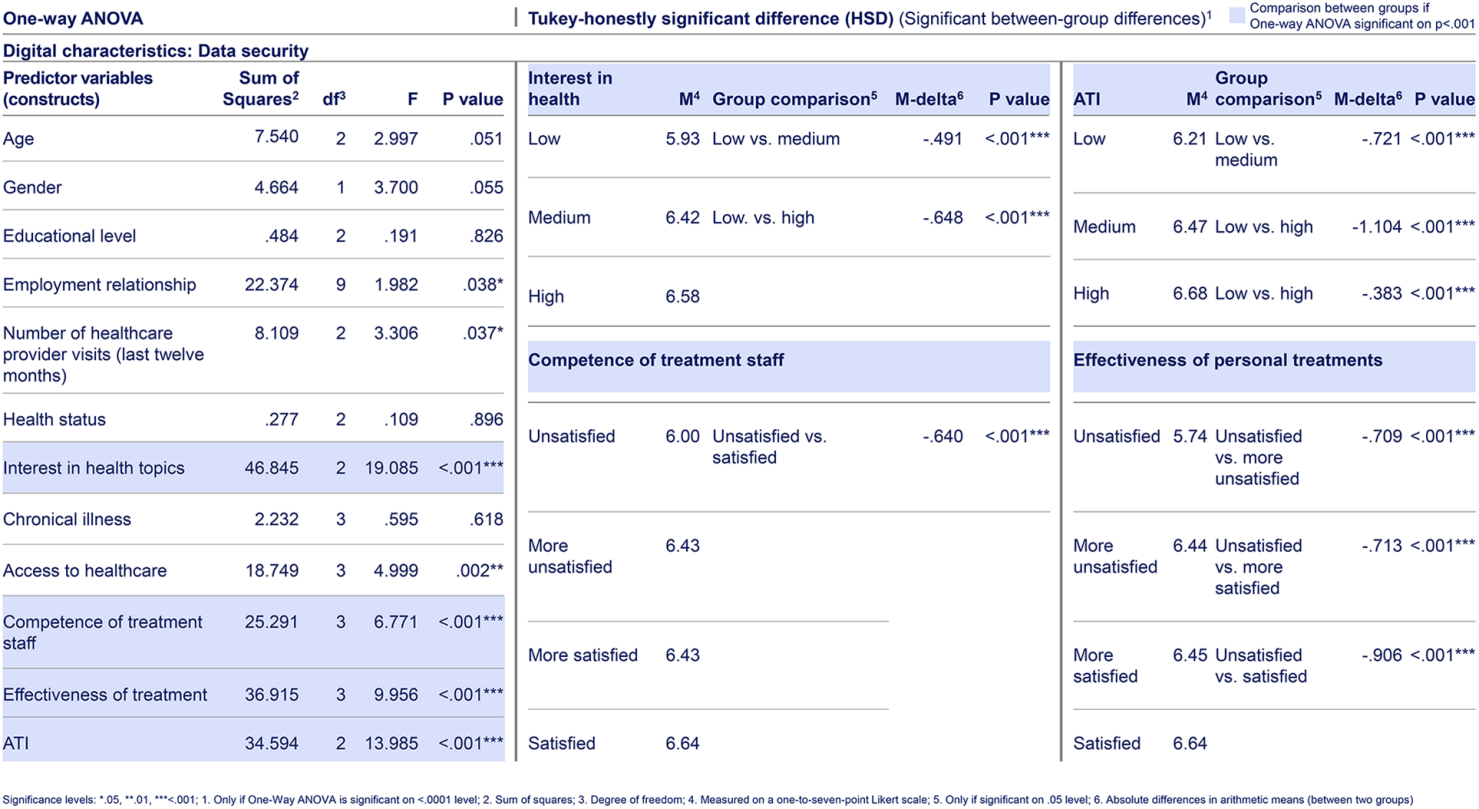
Selected web-survey one-way ANOVA and Tukey-HSD test result of citizens’ for “digital characteristics.” Based on primary data collected via a web-survey including n = 1289 German citizens, using a one-to-seven-point Likert scale, with one being “very unlikely” and seven “very likely.” The web-survey included closed-ended questions based on a literature review and primary data from semi-structured qualitative interviews. For each predictor variable (construct) per dependent variable, respectively answer item, a Tukey-HSD test was conducted, if the one-Way ANOVA test indicated a highly significant difference between citizen groups in that predictor variable (construct). For the digital characteristic “data security,” there is no significant difference in appreciation between citizens’ demographics, including “age,” “gender,” “educational level,” and “employment relationship” groups.

In regard to the first research question (added values), citizens primarily perceive an added value from digital health ecosystems through the online management of health needs, rather than from “personalized health services” (see Table 1). The latter is highly significantly greater for citizens with an above-average “number of healthcare provider visits (last 12 months)” in contrast to citizens without visits (*p* < .001, M-delta = .640, d = .38) (see Table 3).

Regarding the second research question (services and interactions), the likelihood of citizens demanding services and interactions in digital health ecosystems increases highly significantly with greater “health interest” and “ATI” (for both across all services and interactions at *p* < .001; with seven times d ≥ .5 < .8, 14 times d ≥ .8 ≤ 1, and seven times d > 1 for “health interest,” as well as with four times d < .5, 23 times d ≥ .5 < 0.8, and one time d ≥ .8 ≤ 1 for “ATI”. Citizens consistently demand services and interactions that increase the convenience through the online management and access of health needs along the health journey, as well as value online services that involve health professionals in the interaction. “Peer-to-peer” interactions have a lower priority (see Table 1). Younger citizens (“age” < 30 versus ≥60) show significantly higher interest in more innovative services and interaction options beyond the online management and access of health needs along the health journey, including personalized recommendations (e.g. “training offerings” *p* < .001, M-delta = .622, d = .38), online options (e.g. “healthcare provider chat boot” *p* < .001, M-delta = .726, d = .43), as well as “qualification for discounted insurance rates,” and “payer and insurer chat boot” (both at *p* < .001; M-delta = .676, d = 0.39; M-delta = 1.097, d = .63, respectively).

In addition to these overarching results, the web-survey shows the following health journey step dedicated results:

In prevention, citizens show a demand for “activity level storing” services but not for “sharing activities with peers.” Among citizens with above-average “number of healthcare provider visits (last 12 months)” compared to those without healthcare provider visits, there is a significantly higher level of appreciation of “personalized prevention recommendation,” “activity level storing,” and “automatic health status monitoring” services (all three at *p* < .001; M-delta = .154, d = .58; M-delta = .890, d = .49; M-delta = 1.002, d = .55, respectively).

Services and interactions that enable a more convenient access to diagnosis take top priority for citizens. Online diagnosis services with less involvement from health professionals are given lower importance (e.g. “automatic symptom checker,” “advice from peers”), while services involving interactions with health professionals consistently receive higher than the m ratings (e.g. “second healthcare provider opinion for diagnosis” and “online healthcare providers’ information about symptoms”). “Online health history/data exchange” and “central health data storage” are particularly relevant for citizens with above-average “number of healthcare provider visits (last 12 months)” compared to those without such visits (both at *p* < .001; M-delta = .959, d = .59; M-delta = .985, d = .58, respectively) (see Table 4).

Also, regarding treatment and payment services, citizens seek for convenience. Exemplary, top-rated services and interactions are “receiving and extending e-prescriptions” and “(re-)ordering of medications” as well as “transparency on own insurances across policies” and “processing of reimbursement payments.” “Qualification for discounted insurance rates” (M = 4.63) receives an above m rating, which highly significantly increases for the group with the best versus worst perceived “health feeling” (*p* < .001, M-delta = .779, d = .42) (see Table 4).

Considering the third research question (digital characteristics), across services and interactions as well as along the health journey, citizens appreciate digital characteristics that relate to data and privacy protection (see Tables 2 and 5). Also, they value “technical stability” (see Table 2).

Regarding the fourth research question (health service-providers), citizens, regardless of their personal characteristics, primarily expect “public payers” (M = 4.29, SD = 1.99) and also “governmental institutions” (M = 3.43, SD = 1.77) as health service-providers (see Table 2). Non-health-related private sector companies receive the lowest ratings (see Table 2).

## Discussion

The fact that citizens primarily perceive an added value from managing health services online may has been reinforced during the Covid-19 pandemic, as many citizens used digital health apps for the first time and do not want to give up the experienced increase in convenience going-forward.^19^

In terms of services and interactions, citizens’ lower demand for more innovative services has remained unchanged since 2015, despite technological advances,^60^ challenging the thesis that “patients of the future will expect artificial intelligence supported health services,”^15^ potentially driven by four reasons: first, some citizens may have felt overwhelmed by the need to use digital health apps during the Covid-19 pandemic, which may discourage them from adapting even more innovative services and interaction options ^⁠.^^7^ Second, citizens may assume that German health sector stakeholders lack the ability to implement more innovative services and interaction options that are also convenient to use, as they are lagging digital competencies compared to other European countries.^85,86^ Third, more innovative services and interaction options require constant uploads of personal data (e.g. hours slept, steps walked), which are often inconvenient to provide due to required manual data entries.^87^ In line, a Finnish study found that only 16% of activity tracking app users and 9% of heart rate monitor or sport watch owners use these devices frequently.^88^ Lastly, some may question the clinical validity of digital health apps.^47,89^

The nuance, that citizens with a higher “number of healthcare provider visits (last 12 months)” value innovative services and interactions more, is likely because their more frequent interactions with healthcare providers involve sharing personal health data. This creates an expectation of receiving something in return,^52^ e.g. in terms of faster recovery through personalized health. This is supported by citizens being more willing to provide data temporarily during an illness.^25^

“Participant 11 ‘I would appreciate some form of return for this. […] Meaning, when I provide my data, I would not expect generalized offerings anymore’.”

In more detail, there are interesting topics regarding citizens’ demanded services and interactions for discussion along the health journey:

Citizens who are more affected by “permanent/chronic diseases” and have a higher “number of healthcare provider visits (last 12 months)” may be more open toward preventative services, given their higher responsibility for maintaining health and likelihood to benefit from these.^90^ The desire for an increased convenience to access diagnostic services emphasizes the need to further expand the functionalities and adaptation of an electronic health record (EHR) in Germany, which is still in its infancy status,^52^ with a low adaptation level.^91^ EHRs, when avoiding “silo-approaches,”^92^ play a critical role ^⁠^^7,24,33^ in standardizing data storage and exchange between health service-providers, as well as improving the interaction convenience with citizens.^38,93^ Other health systems have shown that this may require investments from governmental policymakers in a central data platform with common data definitions, the technical infrastructure of public healthcare providers and payers, as well as policies mandating mandatory usage.^94^ The low appreciation of “peer-to-peer” interactions may be driven by citizens concerns regarding the credibility of health information, potential reactions from peers to their health data,^95^ and inaccurate health assessments.

“Participant 2 ‘I would search for the experience of peers, however, would not make any self-diagnosis based on it—given diagnosis decisions like this I want to have been done by trained professionals. I even would not trust a self-diagnosis based on the online input of healthcare providers, as I would be afraid to have misinterpreted my own symptoms’.”

Citizens seeking interactions including the involvement of health professionals show that they only want to be empowered to a certain extent,^95^ as they will remain laypersons in health topics.^15^ Instead, digital health ecosystems should be positioned as digital channels that support the citizen–healthcare provider partnership, allowing them to partner as digital companions.^36^ The higher appreciation of online treatment options by the two citizen groups “aged” under 30 and with a higher level of “ATI” aligns with Radó^96^ but extends the view of a study conducted in Australia by demonstrating that online options are relevant beyond prevention contexts,^97^ which may hold particularly true for “less urgent” medical conditions.^⁠.^^7^ The reason could be attributed to the fact that the two citizen groups are better equipped with enabling technologies ^⁠^^8,41,98^ and encounter fewer technical barriers when incorporating these technologies into their daily lives,^25,34,45^ requiring less support.^33,41,43,45,96^ Additionally, younger citizens are more accustomed to instant (video) messaging in non-health contexts^45,95^ and desire the same immediacy for their health interactions. However, health service-providers should establish realistic expectations regarding response times,^45^ as younger citizens are often referred to as “technology optimists” and may be disappointed if their expectations of digital solutions are not met ^⁠.^^6^ Regarding payment services, an opportunity arises for insurers among citizens who have a better “health feeling” and a younger “age,” as they show interest in the “qualification for discounted insurance rates.” Insurers can improve their client risk portfolio by tracking and rewarding healthy activities (e.g. hours slept, steps per day). Penalizing citizens with less healthy lifestyles through maluses should be avoided, as it may discourage their support of the “qualification for discounted insurance rates.”

“Participant 1 ‘Which means I would be afraid of getting ‘maluses’, and that I need to pay over-proportional for my health insurance […]’.”

Regarding citizens’ attitudes toward digital characteristics, the strong request for data protection is consistent with prior research.^95,99^ Negative experiences with digital health apps may have increased citizens’ attention to data protection, as some health service-providers track data for third parties^100^ or process user data contrary to privacy policies.^101^ Addressing citizens’ concerns could involve holding all health service-providers accountable for data protection, rather than solely the orchestrator^102^ as well as granting full access to all stored data, including the right to delete^24^ and “individual data access control” for each health service-provider.

German citizens mandate public sector institutions as orchestrator of digital health ecosystems, whereas in other countries, private insurers have successfully established themselves as orchestrators.^31^ Their success may be attributed to the fact that public sector institutions lack capacity,^35,103^ funding strategies, and focus^104^ or the funds itself to finance the necessary investments.^91^ Public sector institutions in Germany may still be mandated, as citizens consistently prioritize data protection, which they more associate with “public authorities.”^7,33,105^ “Governmental institutions” may also be best positioned to establish common application programming interfaces (APIs)^106^ and EHR standards that enable the integration of services across health service-providers.^47^

The low rating of technology-related private sector companies (such as Google, Facebook, Instagram, Apple) as orchestrator challenges Simpson et al.’s view, who suggest a shift toward free-market dynamics to improve health services.^93^ While technology companies may be technically well-positioned and citizens accustomed to sharing data with them, this may not be as applicable in health contexts.^95^

Reflecting on the international applicability of the research study's findings, they may be particularly relevant for countries with health system structures similar to Germany along the four indicators^53^ (see “Introduction” chapter for details and exemplary countries). Regarding the indicator financing, which often varies across countries,^54,56^ prior research studies (including one focused on the US) have called out the need to adopt reimbursement schemes for digital health (ecosystem) services and interactions as they are currently insufficiently covered.^33,47^ This also applies to the German health insurance system, especially to the mandatory health insurance offered by public payers.^107,108^ Reimbursement coverage impacts citizens’ attitudes, as the willingness to use digital health services and interactions decreases if they need to be paid out-of-pocket.^109^ Additionally, the “digital maturity” of service organizations (e.g. in terms of interoperability, EHR implementation status, accessing and linking datasets, data sharing, data catalogs) may influence citizens’ attitudes directly or the ability to satisfy them.^50,52,54^ In this regard, across OECD countries, the German health system can be considered a laggard.^55^ Similarly, prior research studies with worldwide samples have identified a lacking centralized data access combined with an infancy EHR integration status as the main barriers to scale digital health ecosystems.^24,37,47^ This might increase the applicability of the research study's findings internationally, as most countries have similar digital prerequisites.

Beyond the four indicators, the international applicability of the findings may depend on factors such as culture aspects, countries’ health risks, and citizens’ personal characteristics (e.g. demographics, health status, “VPSI,” and “ATI”). Consequently, findings may have particularly relevance for countries with citizens having similar personal characteristics to the ones of the sample in this research study. Following the research study's findings, in countries with, on average, citizens being younger, visiting practitioners more frequently, or having a higher “ATI” compared to Germany, the demand for more innovative services and interactions might be higher overall. This could potentially result in greater challenges for other stakeholder groups, such as governmental policymakers and health service-providers, to satisfy citizens’ attitudes.

In line with the findings of this research study, Morley and Floridi (2020) have shown that UK citizens have an attitude to strengthen the relationship with their practitioners through digital health ecosystems but do not want to replace interactions with practitioners, rather, they aim make health decisions on their own.^36^ Similarly, Portuguese senior citizens have a higher acceptance for digital health services and interactions in a prevention, rather than a treatment context.^47^ As shown by Zanaboni and Fagerlund (2020), Norwegian citizens have similar attitudes to German citizens’, primarily demanding convenience increasing digital health-related services and interactions (e.g. “electronic booking of appointments” followed by “electronic prescription renewal service”), rather than innovative one as well as accept lower practitioner involvement for “non-clinical inquiries” only. In-line, Norwegian citizens’ highest rated added value was convenience related (time savings).^71^

Beyond single country-focused research studies, those with rather global samples also indicate that citizens’ attitudes are rather aligned and consistent to the findings of this research study. For example, several research studies have shown a high appreciation of centralized and integrated digital access to all health-related digital and in-person services.^25,33,47^ Additionally, digital characteristics such as data security and safeguarding privacy are relatively universally valued by citizens.^33,36^ In line with our findings, prior research studies with international samples have confirmed that the digital maturity of citizens (ATI) is a key driver for citizens’ attitudes toward digital health ecosystems, which often varies within each country itself by age groups.^33,45,98^ This might indicate the need to differentiate digital health ecosystems by target groups based on their “ATI,” respectively age. Developing countries may structurally have a lower “ATI” due to a lower penetration of enabling technologies and devices, driven by lagging affordability.^92,103,106^ This might limit the international applicability of the findings for countries with a higher need to improve citizens’ overall well-being.

## Conclusions

### A three-staged approach to establishing user-centered digital health ecosystems

**Figure 4.**
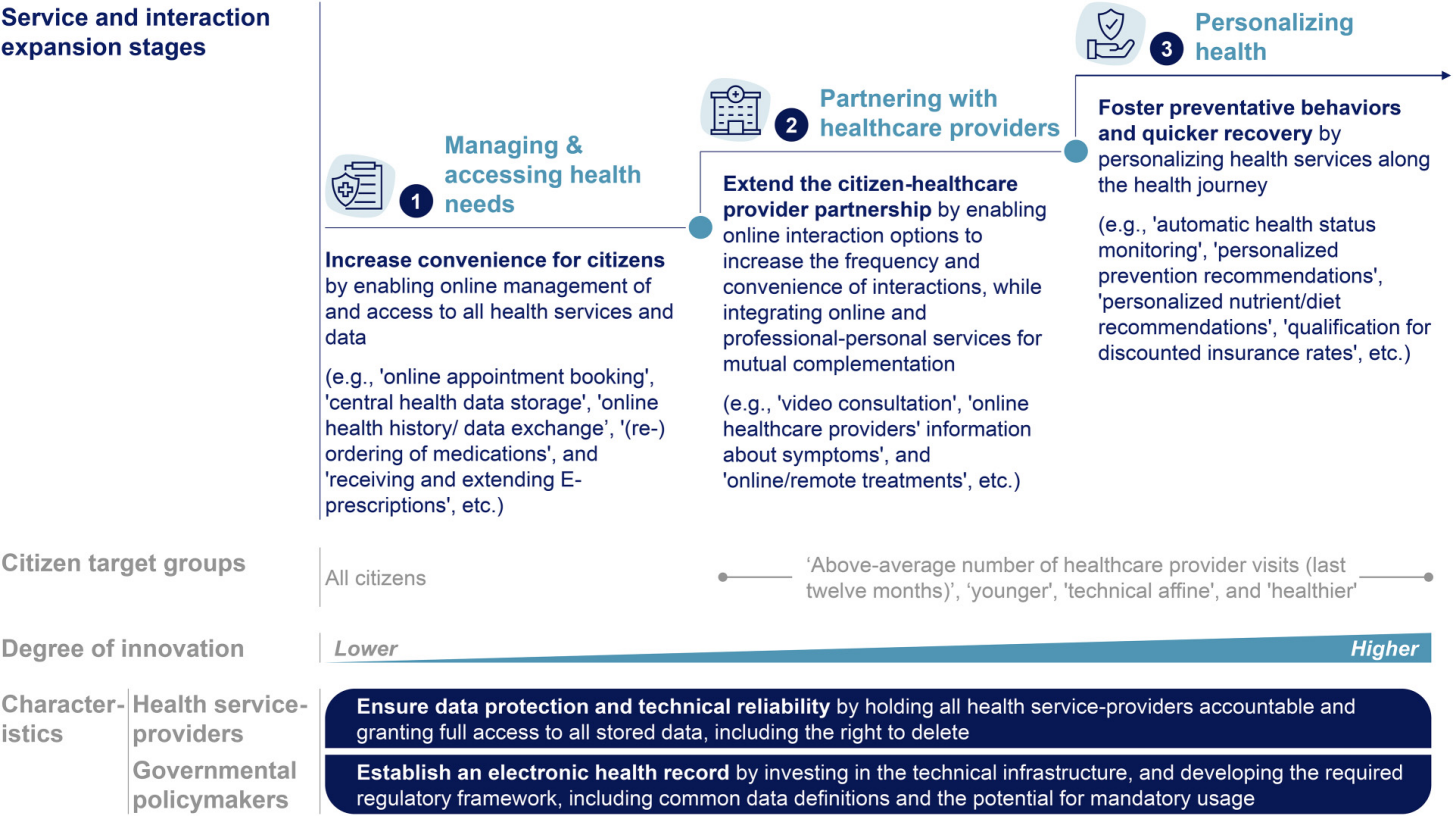
Three-stage approach to establish user-centered digital health ecosystems. Based on three-step sequential mixed method approach including a literature review as well as primary data from n = 15 semi-structured qualitative interview participants and n = 1289 web-survey respondents. Shifting from managing health, partnering with healthcare providers, to personalizing health toward a user-centered digital health ecosystem.

Based on the findings of this study, governmental policymakers and decision-makers at health service-providers may gradually expand digital health ecosystems along the health journey, following three stages that align with citizens’ attitudes to ensure lasting adaptation. This three-staged approach is detailed in Figure 4.

First, the online management of and access to health services and data should be integrated to increase the convenience for citizens. Second, online interaction options should be integrated to complement professional–personal health services, extending the citizen–healthcare provider partnership. Third, services should be personalized to foster preventative behaviors and quicker recovery, requiring a smooth integration of data collection devices (e.g. wearables) to enable automated data transfer.

Overall, health service-providers need to pay attention to data protection and technical stability to gain the trust of citizens. As citizens’ demand services and interactions that are closely linked to the functionalities of an EHR as a key enabler, governmental policymakers should prioritize to promote usage by investing in the technical infrastructure and developing the required regulatory framework, including common data definitions and the potential for mandatory usage.

### Limitations and strengths

The study's important findings are subject to certain limitations.

Firstly, as the main source of primary data collection was conducted online, the findings might be biased toward citizens who have a higher propensity to use online services and interactions. However, the use of a web-based survey allowed for a sizeable sample diversified across various predictor variables (constructs), allowing to generate generalizable findings. Therefore, the literature review did not represent a standalone data source or methodological approach but served as the basis for the thematic analysis of the interviews, sequentially developing the web-survey questionnaire. Thus, the literature review's search strategy was focused (e.g. in terms of data bases) and may not represent a comprehensive “scoping review.”

Secondly, although best efforts to identify relevant a priori codes as answer items, there is a possibility that some might have been missed due to the focused search strategy of the literature review and the limited existing body of research on this topic. To mitigate this potential risk, the codes representing the answer items of the web-survey questionnaire were supplemented with primary data obtained from interviews.

Thirdly, the findings of this research study are based on ratings using Likert scales, which measure citizens’ attitudes. Due to the non-resource constraint primary data collection format, respondents were not required to make trade-offs, resulting in stated preferences not being measured.^68,110^ Given the absence of an established digital health ecosystem in Germany, while leveraging its distinct dual health system, this methodology was considered the most appropriate. It allowed to derive findings from a large sample and to increase the comparability of findings when repeating the study in other countries internationally.^68,69,111,112^

Lastly, the applicability of findings across heath systems in an international context might be constrained to selective countries, given the variety in health system structures (e.g. governance, financing, resources, and service organization) and beyond, including cultural aspects, countries’ health risk factors, citizens’ personal characteristics such as demographics, health status, “VPSI,” and “ATI,” as well as differences in the digital maturity of governmental policymakers and health service-providers.^54^

### Further research inquiries

Drawing from the findings of this study, three directions for further scientific inquiries emerge: first, research studies could further investigate the findings of this research by using stated preference measuring methodologies. This could add value for health service-providers who might want to prioritize certain subsegments of services or interaction options first before scaling. Similarly, the findings could be further investigated by replicating the research study in countries with citizens’ personal characteristics different from Germany or a higher digital maturity among policymakers and health service-providers to facilitate generalization. Second, there is a need for a deeper understanding of citizens’ perspectives regarding the data they are willing to share, the frequency, recipients, and the contextual conditions under which they are comfortable sharing it. Third, the health service-providers’ perspectives should be detailed including their expectations, incentives, and required capabilities.

## Supplemental Material

sj-docx-1-dhj-10.1177_20552076241255929 - Supplemental material for Understanding citizens’ attitudes within user-centered digital health ecosystems: A sequential mixed method methodology including a web-survey Supplemental material, sj-docx-1-dhj-10.1177_20552076241255929 for Understanding citizens’ attitudes within user-centered digital health ecosystems: A sequential mixed method methodology including a web-survey by Robin Huettemann, Benedict Sevov, Sven Meister and Leonard Fehring in DIGITAL HEALTH

sj-docx-2-dhj-10.1177_20552076241255929 - Supplemental material for Understanding citizens’ attitudes within user-centered digital health ecosystems: A sequential mixed method methodology including a web-survey Supplemental material, sj-docx-2-dhj-10.1177_20552076241255929 for Understanding citizens’ attitudes within user-centered digital health ecosystems: A sequential mixed method methodology including a web-survey by Robin Huettemann, Benedict Sevov, Sven Meister and Leonard Fehring in DIGITAL HEALTH

sj-docx-3-dhj-10.1177_20552076241255929 - Supplemental material for Understanding citizens’ attitudes within user-centered digital health ecosystems: A sequential mixed method methodology including a web-survey Supplemental material, sj-docx-3-dhj-10.1177_20552076241255929 for Understanding citizens’ attitudes within user-centered digital health ecosystems: A sequential mixed method methodology including a web-survey by Robin Huettemann, Benedict Sevov, Sven Meister and Leonard Fehring in DIGITAL HEALTH

sj-docx-4-dhj-10.1177_20552076241255929 - Supplemental material for Understanding citizens’ attitudes within user-centered digital health ecosystems: A sequential mixed method methodology including a web-survey Supplemental material, sj-docx-4-dhj-10.1177_20552076241255929 for Understanding citizens’ attitudes within user-centered digital health ecosystems: A sequential mixed method methodology including a web-survey by Robin Huettemann, Benedict Sevov, Sven Meister and Leonard Fehring in DIGITAL HEALTH

sj-docx-5-dhj-10.1177_20552076241255929 - Supplemental material for Understanding citizens’ attitudes within user-centered digital health ecosystems: A sequential mixed method methodology including a web-survey Supplemental material, sj-docx-5-dhj-10.1177_20552076241255929 for Understanding citizens’ attitudes within user-centered digital health ecosystems: A sequential mixed method methodology including a web-survey by Robin Huettemann, Benedict Sevov, Sven Meister and Leonard Fehring in DIGITAL HEALTH
