# Supplementary material for: Understanding citizens’ attitudes within user-centered digital health ecosystems: A sequential mixed method methodology including a web-survey
Source: Digit Health. 2024 May 20;10:20552076241255929. doi: 10.1177/20552076241255929 (PMC11418335; doi:10.1177/20552076241255929)
Supplement: sj-docx-1-dhj-10.1177_20552076241255929 - Supplemental material for Understanding citizens’ attitudes within user-centered digital health ecosystems: A sequential mixed method methodology including a web-survey [file sj-docx-1-dhj-10.1177_20552076241255929.docx]

**Original Research – Supplementary Material 1**

# Understanding citizens’ attitudes within user-centered digital health ecosystems: a sequential mixed method methodology including a web-survey

Robin Huettemann^1,5^, Benedict Sevov^1,6^, Sven Meister^2,3,7^, Leonard Fehring^1,4,8,*^

Affiliations:

1: Faculty of Health, School of Medicine, Witten/Herdecke University, Witten, Germany. *[Primary affiliation]*

2: Healthcare Informatics, Faculty of Health, School of Medicine, Witten/Herdecke University, Witten, Germany. *[Primary affiliation]*

3: Department Healthcare, Fraunhofer Institute for Software and Systems Engineering ISST, Dortmund, Germany.

4: Gastroenterology, HELIOS University Hospital Wuppertal, University Witten/Herdecke, Wuppertal, Germany.

5: ORCID: 0000-0003-3908-3029

6: ORCID: 0009-0000-2959-2394

7: ORCID: 0000-0003-0522-986X

8: ORCID: 0000-0002-3322-3724

* Corresponding author:

**Leonard Fehring**

**Address**

Witten/Herdecke University

School of Medicine

Faculty of Health

Alfred-Herrhausen-Strasse 50

58448 Witten

Germany

Email leonard.fehring@uni-wh.de

Phone +49 157 85520426

## **Supplementary Material 1.** Interviewed guide with open-ended questions for semi-structured qualitative interviews.

The interview guide is structured along six sections:

1. **General information and digital health ecosystem definition**
2. **Added values**
3. **Services and interactions**
4. **Digital characteristics**
5. **Health service-providers**
6. **Citizen personal characteristics/predictor variables**
7. **General information and digital health ecosystem definition**
8. **Added values**

What kind of perceived added values would you expect in a digital health ecosystem?

1. **Services** **and interactions**

What kind of services and interaction options would you demand in a digital health ecosystem?

1. **Digital characteristics**

What digital health ecosystem digital characteristics would be important to you?

1. **Health service-providers**

From which type of health service-provider as orchestrator would you be most likely to use a digital health ecosystem?

1. **Citizen personal characteristics/predictor variables**

How old are you?

Which gender do you identify with?
