# Supplementary material for: Understanding citizens’ attitudes within user-centered digital health ecosystems: A sequential mixed method methodology including a web-survey
Source: Digit Health. 2024 May 20;10:20552076241255929. doi: 10.1177/20552076241255929 (PMC11418335; doi:10.1177/20552076241255929)
Supplement: sj-docx-2-dhj-10.1177_20552076241255929 - Supplemental material for Understanding citizens’ attitudes within user-centered digital health ecosystems: A sequential mixed method methodology including a web-survey [file sj-docx-2-dhj-10.1177_20552076241255929.docx]

**Original Research – Supplementary Material 2**

# Understanding citizens’ attitudes within user-centered digital health ecosystems: a sequential mixed method methodology including a web-survey

Robin Huettemann^1,5^, Benedict Sevov^1,6^, Sven Meister^2,3,7^, Leonard Fehring^1,4,8,*^

Affiliations:

1: Faculty of Health, School of Medicine, Witten/Herdecke University, Witten, Germany. *[Primary affiliation]*

2: Healthcare Informatics, Faculty of Health, School of Medicine, Witten/Herdecke University, Witten, Germany. *[Primary affiliation]*

3: Department Healthcare, Fraunhofer Institute for Software and Systems Engineering ISST, Dortmund, Germany.

4: Gastroenterology, HELIOS University Hospital Wuppertal, University Witten/Herdecke, Wuppertal, Germany.

5: ORCID: 0000-0003-3908-3029

6: ORCID: 0009-0000-2959-2394

7: ORCID: 0000-0003-0522-986X

8: ORCID: 0000-0002-3322-3724

* Corresponding author:

**Leonard Fehring**

**Address**

Witten/Herdecke University

School of Medicine

Faculty of Health

Alfred-Herrhausen-Strasse 50

58448 Witten

Germany

Email leonard.fehring@uni-wh.de

Phone +49 157 85520426

## **Supplementary Material 2.** Reporting of semi-structured qualitative interviews along the ‘Consolidated criteria for reporting qualitative research (COREQ): a 32-item checklist for interviews and focus groups’. ^1^

| **Section and topic** | | **Items** | **Item reporting** |
| --- | --- | --- | --- |
| **Domain 1: Research team and reflexivity** | | | |
| **Personal Characteristics** | *Interviewer/ facilitator* | ***1*** | All interviews were conducted by the same co-author to mitigate any biases that could arise from different interviewers. |
|  | *Credentials* | 2 | The highest academic degree of the interviewer was a ‘Master of Science’ degree. The interview approach was aligned with the further co-authors, who held ‘Professorship’, ‘Medical Doctor’, and ‘Master of Science‘ as the highest credentials. |
|  | *Occupation* | 3 | At the time of the interviews, the interviewer was a full time Ph.D. student, occupied as a research assistant. Further co-authors involved in developing the interview approach were occupied as researchers, physicians, university lectures, and psychology student. |
|  | *Gender* | 4 | Male. |
|  | *Experience and training* | 5 | The interviewer had experience in interviewing from different perspectives: 1) The interviewer previously conducted semi-structured qualitative interviews as part of earlier research projects. 2) In a former role as a consultant, the interviewer received professional expert interview training and conducted several interviews with different senior executives and experts in the health and insurance sectors on varies topics, partially through the expert network ‘alphasights’. In addition, the interview approach was aligned with the experienced co-authors, who had conducted semi-structured qualitative interviews in several peer-reviewed publications. Their experiences and ‘lessons-learned’ have been incorporated in the design. |
| **Relationship with participants** | *Relationship established* | 6 | The first contact with potential participants was via a text message established, either on Facebook, or LinkedIn. All participants have been contacted by the interviewer directly in a written way. |
|  | *Participant knowledge of the interviewer* | 7 | Some participants were aware of the interviewer was performing the interviews as part of his Ph.D. program, as they asked during the introduction section of the interviews. Others may have visited the interviewer's LinkedIn profile, from which they could have derived the interviewer was enrolled in a Ph.D. program. |
|  | *Interviewer characteristics* | 8 | The interviewer’s characteristics or intentions were not addressed during the interview process. However, the interviewer stated the scientific reason of the interviews, the overall research sequence and objective, as well as how the interviews contribute to them. Although not mentioned proactively, when questioned, the interviewer also explained the overarching goal of obtaining a Ph.D. degree. |
| **Domain 2: Study design** | | | |
| **Theoretical framework** | *Methodological orientation and theory* | 9 | The methodology followed a thematic analysis, which is referred to as specific type of content analysis, based on the understanding of Braun and Clarke: ‘[…] the unit of analysis tends to be more than a word or phrase, which it typically is in content analysis.’ ^2^ This approach is both inductive and at the same deductive, allowing for a semi-quantitative analysis of the interviews while investigating connections, patterns, and reasoning.  Overall, the interviews were part of a sequential mixed-method methodology: Interviews were used to develop exploratory insights, which were tested quantitively in the subsequent stage of the research project. |
| **Domain 2: Research team and reflexivity** | | | |
| **Participant selection** | *Sampling* | 10 | Participant sampling was purposive to ensure an equal number of participants across gender and age groups. Potential interview participants did not need to have specific prior knowledge (Supplementary Material 5). |
|  | *Method of approach* | 11 | After the initial contact through written communication, all interviews were conducted in a virtual setup in the format of online video calls, with only the interviewer and one participant present. |
|  | *Sample size* | 12 | 15 participants participated in the qualitative interviews (Supplementary Material 5). |
|  | *Non-participation* | 13 | Some potential participants responded to the initial written contact approach but declined to participate due to limited interest in the topic or time constrains. All those who agreed to be interviewed completed the process. |
| **Setting** | *Setting of data collection* | 14 | Data collection took place through online video calls. Participants were asked to choose a comfortable environment where they could be alone and free from distractions during the interview time, which in most cases was either at home or at work, depending on the time of day. |
|  | *Presence of non-participants* | 15 | No, only the participant and the interviewer were present during the online video calls. |
|  | *Description of sample* | 16 | Please refer to Supplementary Material 5 for details on the personal characteristics of each interview participant, as well as the duration of each interview. The objective was to generate a sample that was equally distributed across age and gender groups. |
| **Data collection** | *Interview guide* | 17 | The interview guide was pre-tested with three participants to ensure the understandability of questions. Their answers were not recorded, respectively considered in the findings of this research. To ensure all participants had the same understating of digital health ecosystems, the definition was clarified at the beginning of each interview.  Each semi-structured qualitative interview followed the same interview guide with open-ended questions, which remained unchanged throughout all interviews. The interviewer might ask follow-up questions within the interview. The interviewer selectively summarized statements to ensure a correct and aligned understanding. Participants spoke freely. |
|  | *Repeat interviews* | 18 | Each participant was interviewed once, and all interviews followed the same structure and interview guide. |
|  | *Audio/visual recording* | 19 | The semi-structured qualitative interviews were voice only recorded, as gestures, volume, facial expressions, and time stamps were not relevant to answering the research questions, protecting participants’ privacy, and creating a more comfortable environment to answer the questions. Participants were informed about and consented to the voice recording before the interviews, respectively recordings started. |
|  | *Field notes* | 20 | No field notes were taken, as the interviews were recorded for transcription. |
|  | *Duration* | 21 | Interviews were held between 20 March and 14 April 2023, with each interview lasting between 16 to 23 minutes. |
|  | *Data saturation* | 22 | Data saturation was not discussed with the participants during or after the interviews. However, as part of the coding process, the two co-authors discussed the repetition of codes to jointly define the point of data, respectively content saturation. This, in return, triggered the discontinuation of interviewing further participants. |
|  | *Transcripts returned* | 23 | Transcripts were not returned to participants because accurate and detailed transcripts could be generated from the voice-only recordings. These transcripts were factually anonymized, meaning to identify the underlying individual would only be possible with disproportionated effort (if possible, at all) ^3^. Transcripts were selectively smoothened when appropriated (e.g., preverbal sentences were excluded such as ‘hm’, ‘äh’, etc.). |
| **Domain 3: Analysis and findings** | | | |
| **Data analysis** | *Number of data coders* | 24 | Interview transcripts were coded by two co-authors who first familiarized themselves with the transcripts, followed by three rounds of coding. First, statements mentioned as relevant by the participants were either assigned to one of the a priori codes or supplemented as distinct new codes. Second, codes were revised, which included adding details, renaming, or merging them. Third, a second co-author reviewed the coding. Codes were either confirmed or discussed until both were confident that the coding accurately represented the interview data. |
|  | *Description of the coding tree* | 25 | Yes, Supplementary Material 3 includes all the code names. The first level of the coding tree links to the research questions: 1) added values, 2) services and interactions, 3) digital characteristics, and 4) health service-providers. Codes related to 2) services and interactions are further clustered along the health journey steps. |
|  | *Derivation of themes* | 26 | As the research followed a sequential mixed method methodology, codes were either identified as part of the literature review (referred to as a priori codes) or derived from the thematic analysis based on the interview transcripts of the semi-structured qualitative interviews. Codes were assigned to statements mentioned as relevant by the participants and either assigned to one of the a priori codes or supplemented as distinct new codes. Supplementary Material 3 specifies if codes were either derived from the literature review (indicated by (‘L’)) or from the semi-structured qualitative interviews (indicated by (‘I’)). |
|  | *Software* | 27 | MAXQDA (Version 2022.4) was used for the data analysis. |
|  | *Participant checking* | 28 | Feedback from the participants was not collected. |
| **Reporting** | *Quotations presented* | 29 | Participant quotations were used to provide examples for the most important identified codes and used to facilitate the discussion. Each quotation is allocated to a unique factually anonymized participant ID and always referenced once when quoting. |
|  | *Data and findings consistent* | 30 | Yes, the findings partially align with previous research and is mentioned in the discussion chapter of the manuscript accordingly. |
|  | *Clarity of major themes* | 31 | Yes, in the results chapter, the key results from the qualitative interviews are presented, including exemplary quotations. In addition, exemplary quotes are used in the discussion chapter to discuss the findings. |
|  | *Clarity of minor themes* | 32 | No. |

References

1. Tong A, Sainsbury P and Craig J. Consolidated criteria for reporting qualitative research (COREQ): a 32-item checklist for interviews and focus groups. *Int J Qual Health Care* 2007; 19: 349–357.

2. Braun V and Clarke V. Using thematic analysis in psychology. *Qualitative Research in Psychology* 2006; 3: 77–101.

3. Meyermann A and Porzelt M. *Hinweise zur Anonymisierung qualitativer Daten. Version 1.1.* 1st ed. Frankfurt am Main: DIPF | Leibniz-Institut für Bildungsforschung und Bildungsinformation, 2014.
