# Supplementary material for: Understanding citizens’ attitudes within user-centered digital health ecosystems: A sequential mixed method methodology including a web-survey
Source: Digit Health. 2024 May 20;10:20552076241255929. doi: 10.1177/20552076241255929 (PMC11418335; doi:10.1177/20552076241255929)
Supplement: sj-docx-3-dhj-10.1177_20552076241255929 - Supplemental material for Understanding citizens’ attitudes within user-centered digital health ecosystems: A sequential mixed method methodology including a web-survey [file sj-docx-3-dhj-10.1177_20552076241255929.docx]

**Original Research – Supplementary Material 3**

# Understanding citizens’ attitudes within user-centered digital health ecosystems: a sequential mixed method methodology including a web-survey

Robin Huettemann^1,5^, Benedict Sevov^1,6^, Sven Meister^2,3,7^, Leonard Fehring^1,4,8,*^

Affiliations:

1: Faculty of Health, School of Medicine, Witten/Herdecke University, Witten, Germany. *[Primary affiliation]*

2: Healthcare Informatics, Faculty of Health, School of Medicine, Witten/Herdecke University, Witten, Germany. *[Primary affiliation]*

3: Department Healthcare, Fraunhofer Institute for Software and Systems Engineering ISST, Dortmund, Germany.

4: Gastroenterology, HELIOS University Hospital Wuppertal, University Witten/Herdecke, Wuppertal, Germany.

5: ORCID: 0000-0003-3908-3029

6: ORCID: 0009-0000-2959-2394

7: ORCID: 0000-0003-0522-986X

8: ORCID: 0000-0002-3322-3724

* Corresponding author:

**Leonard Fehring**

**Address**

Witten/Herdecke University

School of Medicine

Faculty of Health

Alfred-Herrhausen-Strasse 50

58448 Witten

Germany

Email leonard.fehring@uni-wh.de

Phone +49 157 85520426

## **Supplementary Material 3.** Questionnaire with closed-ended questions for the quantitative web-survey, including the code names and descriptions as well as grouping of predictor variables (constructs).

The questionnaire is structured along six sections:

1. **General information and digital health ecosystem definition**
2. **Added values**
3. **Services** **and interactions**
4. **Digital characteristics**
5. **Health service-providers**
6. **Citizen personal characteristics/predictor variables (constructs)**

*Legend*

(L) = A priori code based on literature review

(I) = Code based on interviews

*[Name (a priori) code/answer item]*

*(Grouping)*

1. **Section: General information and digital health ecosystem definition**
2. **Section: Added values**

**How likely do you to expect the following added values in a potential digital health ecosystem (digital health app)?**

*Individual rating of each answer item*

- Likert scale 1-7: ‘Very unlikely’ to ‘very likely’ plus eighth ‘no answer preferred’ option

1. Central coordination of own health data (e.g., treatment history, insurance data) and of all health needs (e.g., treatments)

*[Central coordination of health needs (L)]*

1. Transparency across all available health services (e.g., physiotherapists)

*[Transparency across all available health services (I)]*

1. Quick access to health services (e.g., making an appointment)

*[Quick access to health needs (L)]*

1. Personalized health services (e.g., intake of drugs)

*[Personalized health services (L)]*

1. More effective self-recovery (e.g., quicker recovery)

*[More effective self-recovery (L)]*

1. Healthier lifestyle (e.g., tips to adjust lifestyle)

*[Healthier lifestyle (L)]*

1. Other/free text
2. **Section: Services** **and interactions**

**How likely are you to demand the following services and interaction options in a potential digital health ecosystem (digital health app)?**

*Individual rating of each answer item*

- *Likert scale 1-7: ‘Very unlikely’ to ‘very likely’ plus eighth ‘no answer preferred’ option*

***Prevention***

1. Activity level storing (e.g., blood pressure, heart rate)

*[Activity level storing (L)]*

1. Automatic monitoring of health status (e.g., analyses of blood pressure, heart rate, and automatic warnings in case of abnormalities etc.)

*[Automatic health status monitoring (L)]*

1. Personalized training offerings (e.g., training plans, coaching, online/personal courses)

*[Personalized training offerings (L)]*

1. Personalized nutrient/diet recommendations (e.g., recommended calorie intake, nutrition information)

*[Personalized nutrient/diet recommendations (L)]*

1. Personalized recommendations for prevention activities (e.g., cancer protection, back exercises)

*[Personalized prevention recommendations (I)]*

1. Sharing activities with peers (e.g., sharing kilometers run)

*[Sharing activities with peers (L)]*

***Diagnosis***

1. Healthcare provider search/selection (e.g., based on the evaluations of others)

*[Healthcare provider search/selection (L)]*

1. Central storage of health data (e.g., past diseases, laboratory results, insurance data)

*[Central health data storage (L)]*

1. Online appointment booking (e.g., based on capacity)

*[Online appointment booking (L)]*

1. Online exchange of health history/data (e.g., check-in for personal appointments, first time visit at doctors, e-referrals)

*[Online health history/data exchange (I)]*

1. Receiving more than one healthcare provider recommendation (e.g., based on information provided online)

*[Second healthcare provider opinion for diagnosis (I)]*

1. Video-calls with healthcare providers for diagnosis (e.g., based on the own description)

*[Video consultation (L)]*

1. Search for healthcare providers' information about symptoms online (e.g., in technical paper)

*[Online healthcare providers’ information about symptoms (L)]*

1. Search for experiences of other citizens about symptoms online (e.g., blogs, forums)

*[Advice from peers (L)]*

1. Sharing own experiences about symptoms online with other citizens (e.g., in blogs, forums)

*[Sharing experience with peers (L)]*

1. *Control question:* Please tick the first option to the left
2. Automatic symptom checker (e.g., recommendations for further treatments)

*[Automatic symptom checker (L)]*

***Treatment***

1. Chat-hours with healthcare providers for follow-up questions (e.g., chatting directly with healthcare providers during certain times)

*[Chat-hours with healthcare providers (I)]*

1. Chat boot (e.g., chatting with an AI, providing instance response)

*[Healthcare provider chat boot (I)]*

1. Online/remote treatment (e.g., virtual group or individual sessions with physiotherapists)

*[Online/remote treatment (L)]*

1. Receiving and extending e-prescriptions (e.g., prescription extension without going to the doctor in person)

*[Receiving and extending e-prescriptions (L)]*

1. (Re-)ordering of medications (e.g., access to an online pharmacy including shipping)

*[(Re-)ordering medication (L)]*

1. Reminder for medication intake (e.g., smartphone notifications)

*[Reminder for medication intake (L)]*

***Payment***

1. Communication portal with payers and insurers (e.g., chat-function including file exchange and chat history)

*[Communication portal with payers and insurers (I)]*

1. Chat boot (e.g., chatting with an AI, providing instance response)

*[Payer and insurer chat boot (I)]*

1. Processing of reimbursement payments (e.g., online submission of claims)

*[Processing of reimbursement payments (I)]*

1. Closing of on-demand (temporary) insurances (e.g., additional health insurance during holidays/travel)

*[On-demand (temporary) insurance contracts (I)]*

1. Qualification for discounted insurance rates (e.g., monthly financial rewards for an active lifestyle, like amount of steps)

*[Qualification for discounted insurance rates (I)]*

1. Transparency on own insurance across policies (e.g., private, and public policies)

*[Transparency on own insurance across policies (I)]*

1. Other/free text
2. **Section: Digital characteristics**

**How important are the following digital characteristics in a potential digital health ecosystem (digital health app) to you?**

*Individual rating of each answer item*

- *Likert scale 1-7: ‘Very unimportant’ to ‘very important’ plus eighth ‘no answer preferred’ option*

1. Integration of offline/in person offerings

*[Integration of online/personal offerings (L)]*

1. Quick access to the personal relevant offerings (e.g., personalized pre-filtering of the relevant data)

*[Quick access to relevant personal offerings (L)]*

1. Data security (e.g., coverage against unintended external access of third parties)

*[Data security (L)]*

1. Safeguarding privacy (e.g., trust in the data usage)

*[Safeguarding privacy (L)]*

1. Data access control (e.g., selection of data access by individual provider)

*[Individual data access control (I)]*

1. Single log-in to access all offerings (e.g., accessing all services with one single log-in)

*[Single log-in to access all offerings (I)]*

1. Motivating to pursue a healthier lifestyle (e.g., gamification and re-minder/notifications)

*[Motivating (L)]*

1. Collaboration across devices

*[Collaboration across devices (L)]*

1. Appealing app design

*[Appealing app design (L)]*

1. Intuitive use of app

*[Ease of use (L)]*

1. Quick technical support

*[Quick technical support (L)]*

1. Clear description and limited number of required data (at registration)

*[Clear description and limited number of required data (L)]*

1. Technical stability

*[Technical stability (L)]*

1. Other/Free text
2. **Section: Health service-providers**

**How much would you trust the following actors to successfully develop a potential digital health ecosystem, including a digital health app and data storage?**

*Individual rating of each answer item*

- *Likert scale 1-7: ‘No trust’ to ‘high level of trust’ plus eighth ‘no answer preferred’ option*

1. Governmental institutions (e.g., Federal Ministry of Health)

*[Governmental institutions (L)]*

1. Statutory health insurance companies (e.g., Techniker Krankenkasse, AOK)

*[Public payers (L)]*

1. Private insurers (e.g., Allianz, Axa)

*[Private insurers (L)]*

1. Private healthcare providers (e.g., hospitals in private ownership)

*[Private healthcare providers (L)]*

1. Public healthcare providers (e.g., hospitals in public, or church ownership)

*[Public healthcare providers (I)]*

1. Start-ups in the healthcare sector (e.g., Ottonova)

*[Start-ups (L)]*

1. Pharmaceutical companies (e.g., Bayer)

*[Pharmaceuticals companies (L)]*

1. Technology companies (e.g., Google, Facebook, Instagram, Apple)

*[Technology companies (I)]*

1. Other/Free text
2. **Section: Citizen personal characteristics/Predictor variables (constructs)**

***Demographics***

**How old are you?** [‘Age’]

- Free text, in years *(Group 1: <30; Group 2: ≥30<60; Group 3: ≥60)*

**Which gender do you identify with?** [‘Gender’]

*Single option to choose*

- Female (Group: 1)
- Male (Group: 2)
- Diverse (no group, as answer was not chosen by any respondent)
- No answer preferred

**What is your highest level of education?** [‘Education level’]

*Single option to choose*

- Secondary school *(Group: Non-academic education)*
- Apprenticeship/ Training *(Group: Non-academic education)*
- A-level *(Group: Non-academic education)*
- Bachelor *(Group: Academic education)*
- Master’s degree/Diploma *(Group: Academic education)*
- PhD/Professorship *(Group: Academic education)*
- No degree *(Group: Non-academic education)*
- No answer preferred *(Group: Non-academic education)*

**In which employment situation are you currently in?** [‘Employment relationship’)

*Single option to choose*

- Pupil
- Student
- Employed full-time
- Working part-time
- Self-employed
- Public sector
- Retired
- Household tasks/ parental leave
- Seeking for work
- Permanently ill
- No answer preferred

*(No grouping)*

***Health status***

**How many times have you seen a doctor in the last twelve months? (This includes hospitalizations and emergency rooms, but NO dental appointments)?** *[‘number of healthcare provider visits (last twelve months)’]*

- Free text, in #
- No answer preferred

*(Group 1: 0; Group 2: Below average; Group 3: Above average)*

**In general, how would you describe your state of health?** [‘Health feeling’]

*Single option to choose*

- Excellent *(Group: 1)*
- Very good *(Group: 1)*
- Good *(Group: 2)*
- Fair *(Group: 3)*
- Poor *(Group: 3)*
- No answer preferred

**How interested are you in health topics?** [‘Health interest’]

*Single option to choose*

- Extremely interested *(Group: 1)*
- Very interested *(Group: 1)*
- Moderately interested *(Group: 2)*
- Slightly interested *(Group: 3)*
- Not interested *(Group: 3)*
- No answer preferred

**Do you or a close family member currently have a permanent or chronic illness? (e.g., parents, spouse, children, sister/brother, grandparents)?** *[‘Permanent/chronic disease’]*

Single option to choose

- Yes, myself
- Yes, someone in my environment
- Yes, myself & someone in my environment
- No
- No answer preferred

*(No grouping)*

***Vienna Patient Satisfaction Inventory (‘VPSI’)***

*Individual rating of each answer item*

- Dissatisfied
- Rather dissatisfied
- Rather satisfied
- Satisfied

*(No grouping)*

**Vienna Patient Satisfaction Inventory (‘VPSI’): Access to treatment: How satisfied or dissatisfied are you ...** *[‘Access to personal treatments’]*

1. …with, the opening hours of your treatment site?
2. ...with, the waiting times for an appointment at your treatment site?
3. …with, the time it takes you to get from where you live to your common treatment site?
4. ...with, the waiting times at the treatment site before an appointment?
5. ...with, the how long it takes to get mental support?

**Vienna Patient Satisfaction Inventory (‘VPSI’): Competence of treatment staff: How satisfied or dissatisfied are you ...** *[‘Competence of treatment staff’]*

1. …with, how well do treatment teams understand your problems?
2. …with, the level of expertise that treatment teams have for your condition and its treatment?

**Vienna Patient Satisfaction Inventory (‘VPSI’): Effectiveness of treatment: How satisfied or dissatisfied are you ...** *[‘Effectiveness of personal treatments’]*

1. …with, how the treatment improves your health?
2. …with, how the treatment helps you cope better with your problems?
3. …with, how well the treatment meets your expectations?

***Affinity for Technology Interaction Scale (‘ATI’)***

**Please indicate the degree to which you agree/disagree with the following statements.** *[‘ATI’]*

*Individual rating of each answer item*

- Completely disagree *(Group: 1)*
- Largely disagree *(Group: 1)*
- Slightly disagree *(Group: 2)*
- Slightly agree *(Group: 2)*
- Largely agree *(Group: 3)*
- Completely agree *(Group: 3)*

1. ‘I like to occupy myself in greater detail with technical systems.’
2. ‘I like testing the functions of new technical systems.’
3. ‘I predominantly deal with technical systems because I have to.’
4. ‘When I have a new technical system in front of me, I try it out intensively.’
5. ‘I enjoy spending time becoming acquainted with a new technical system.’
6. ‘It is enough for me that a technical system works; I don’t care how or why.’
7. ‘I try to understand how a technical system exactly works.’
8. ‘It is enough for me to know the basic functions of a technical system.’
9. ‘I try to make full use of the capabilities of a technical system.’
