# Supplementary material for: Understanding citizens’ attitudes within user-centered digital health ecosystems: A sequential mixed method methodology including a web-survey
Source: Digit Health. 2024 May 20;10:20552076241255929. doi: 10.1177/20552076241255929 (PMC11418335; doi:10.1177/20552076241255929)
Supplement: sj-docx-4-dhj-10.1177_20552076241255929 - Supplemental material for Understanding citizens’ attitudes within user-centered digital health ecosystems: A sequential mixed method methodology including a web-survey [file sj-docx-4-dhj-10.1177_20552076241255929.docx]

**Original Research – Supplementary Material 4**

# Understanding citizens’ attitudes within user-centered digital health ecosystems: a sequential mixed method methodology including a web-survey

Robin Huettemann^1,5^, Benedict Sevov^1,6^, Sven Meister^2,3,7^, Leonard Fehring^1,4,8,*^

Affiliations:

1: Faculty of Health, School of Medicine, Witten/Herdecke University, Witten, Germany. *[Primary affiliation]*

2: Healthcare Informatics, Faculty of Health, School of Medicine, Witten/Herdecke University, Witten, Germany. *[Primary affiliation]*

3: Department Healthcare, Fraunhofer Institute for Software and Systems Engineering ISST, Dortmund, Germany.

4: Gastroenterology, HELIOS University Hospital Wuppertal, University Witten/Herdecke, Wuppertal, Germany.

5: ORCID: 0000-0003-3908-3029

6: ORCID: 0009-0000-2959-2394

7: ORCID: 0000-0003-0522-986X

8: ORCID: 0000-0002-3322-3724

* Corresponding author:

**Leonard Fehring**

**Address**

Witten/Herdecke University

School of Medicine

Faculty of Health

Alfred-Herrhausen-Strasse 50

58448 Witten

Germany

Email leonard.fehring@uni-wh.de

Phone +49 157 85520426

## **Supplementary Material 4.** Reporting of quantitative web-survey along 30 item ‘Checklist for Reporting Results of Internet E-Surveys (CHERRIES)’. ^1^

| **Section and topic** | | **Items** | **Item reporting** |
| --- | --- | --- | --- |
| **Design** | *Describe survey design* | 1 | The objective was to obtain a sample size of >1,000 respondents, distributed relatively equally across citizen groups with ‘academic’ versus ‘non-academic’ education, ‘age’ groups (<30, ≤30>60, ≥60 years old), and ‘gender’. The sample was a convenience sample. The questions were closed-ended, with predefined answer items. Each answer item is referred to as code and represents one dependent variable, which is either derived from the literature review or semi-structured qualitative interviews. Respondents were asked to rate each depended variable on a one-to-seven-point Likert scale. To avoid pressuring respondents to answers all questions, an eighth ‘no answer preferred’ option as well as a free-text option were added. This was except for questions related to the ‘VPSI’ ^2^ and ‘ATI’ ^3^ constructs as their underlying item scales are validated by prior research. |
| **IRB (Institutional Review Board) approval and informed consent process** | *IRB approval* | 2 | The interview process meets ethical standards, and the ‘Ethics Committee of the Witten/Herdecke University’ (No. S-213/2022) did not raise an objection regarding ethical and ‘General Data Protection Regulation’ (GDPR) concerns. |
|  | *Informed consent* | 3 | Before starting the web-survey, respondents were provided with transparency regarding the amount of questions, the research purpose, and topics covered. They actively indicated their agreement, by checking a virtual ‘tick-mark,’ signifying their voluntary participation and their consent to the data protection strategy. This strategy included details on anonymization, data processing, and data storage practices, which were readily accessible to them. |
|  | *Data protection* | 4 | The data collection, storage, and analysis approaches as part of the web-survey are in line with the German data protection laws. Respondents’ anonymity was ensured at a level of k≥5. Questions with less than five responses were deleted to prevent any potential identification of individual respondents. No personal data, such as name, date of birth, weight, domicile, household size, etc., were collected. The data collection was conducted by using a university license of the software ‘Lime Survey 5.0’.  Prior to the participation, all respondents were provided with written information about the data storing approach, data privacy protection measures, and the scope of the research. |
| **Development and pre-testing** | *Development and pre-testing* | 5 | Prior beginning the field data collection, ten respondents tested the web-survey for technical stability, design, and understandability to ensure a smooth field study. Their responses were not included part of the final data sample. |
| **Recruitment process and description of the sample having access to the questionnaire** | *Open survey versus closed survey* | 6 | The web-survey was accessible for everyone with internet access between 22 May and 30 June 2023. Personalized survey-links or passwords were not used. |
|  | *Contact mode* | 7 | Respondents were contacted through Facebook and LinkedIn, to ensure a sample with diverse backgrounds. Additionally, a panel data provider (similar to Bidmon and Terlutter ^4^) supported the data collection process. |
|  | *Advertising the survey* | 8 | There was no advertising involved. The mail shared by the provider with potential respondents was presented in an informative context, emphasizing voluntary participation. |
| **Survey adminis-tration** | *Web/E-mail* | 9 | The web-survey link was shared via Facebook, LinkedIn, and mail through a panel data provider. Responses were automatically tracked using a unique ID number per respondent, assigned to each potential responded who clicked on the web-survey link. Therewith, progress and timing of each response was tracked on an individual ID level. |
|  | *Context* | 10 | As the web-survey link was distributed via social media and mail, it may have led to a self-selecting sample, consisting of individuals who are generally more open to and experienced in using technologies, online services, and sharing personal data with others. Consequently, the potential of digital health ecosystems, as defined in this study, might be overemphasized compared to citizens who are less digitally or technically inclined. To account for this potential bias, we explicitly assessed the technical affinity of respondents as a predictor variable construct using the established ‘Affinity for Technology Interaction’ scale. |
|  | *Mandatory/ voluntary* | 11 | The web-survey was voluntary for every respondent, with the option to terminate at any progress stage. In case of termination, before answering all questions and submitting, none of their answers were considered. |
|  | *Incentives* | 12 | Depending on the access link used, certain respondents were offered a monetary incentive of 1.60 Euros, while participation remained voluntary at any times. |
|  | *Time/Date* | 13 | Data were collected between the 22 May and 30 June 2023. |
|  | *Randomi-zation of items or questionnaires* | 14 | The order of items and questions remained consistent for all respondents, ensuring that data was collected from each respondent under identical circumstances. |
|  | *Adaptive questioning* | 15 | Adaptive questioning was not used, as the web-survey aimed to gain a comparable perspective across citizens. |
|  | *Number of Items* | 16 | Most pages included one question only, with several answer items to rate for the same question. Two pages included four questions, referring to the citizen personal characteristic categories demographics and health status respectively. |
|  | *Number of screens (pages)* | 17 | Twelve, including the start page with the data protection information. |
|  | *Completeness check* | 18 | A completeness check was conducted. |
|  | *Review step* | 19 | Respondents were able to jump back-and-worth between questions and could change or review their answers. Once submitted, no changes were possible. |
| **Response rates** | *Unique site visitor* | 20 | Unique respondent IP and cookies were used to identify unique visitors and prevent the collection of multiple responses from a single respondent. |
|  | *View rate (Ratio of unique survey visitors/ unique site visitors)* | 21 | Not tracked, as there was no transparency regarding the number of people who viewed the survey link but did not click on it. Tracking of unique user IDs began when individuals clicking on the web-survey link. |
|  | *Participation rate (Ratio of unique visitors who agreed to participate/unique first survey page visitors)* | 22 | 2,375 agreed to participate and proceeded to the first question, which was answered by 2,366 (99.6%). |
|  | *Completion rate (Ratio of users who finished the survey/users who agreed to participate)* | 23 | 95% (before data cleaning). |
| **Preventing multiple entries from the same individual** | *Cookies used* | 24 | The web-survey tool set cookies. |
|  | *IP check* | 25 | The web-survey tool allowed for each IP address a one-time participation only. |
|  | *Log file analysis* | 26 | Not used. |
|  | *Registration* | 27 | Not applicable to ensure anonymity and as the web-survey was accessible to everyone. |
| **Analysis** | *Handling of incomplete questionnaires* | 28 | All questionnaires not submitted (last page) and questionnaires with ‘many unanswered questions’ (chose ‘no answer preferred’ option), were treated as incomplete and were not included in the statistical evaluation. ‘Many unanswered questions’ was defined based on the 10% threshold of respondents who chose the ‘no answer preferred’ option. This threshold in absolute terms was determined after reviewing the first 1,000 complete responses with correct control question answers. Respondents who answered more often with ‘no answer preferred’ than this absolute threshold were excluded. The established threshold was then applied to all subsequent respondents for exclusion. |
|  | *Question-naires submitted with an atypical timestamp* | 29 | The data were filtered to exclude respondents who did not take a reasonable amount of time to answer the questions. ‘Reasonable time’ was defined as taking more time to answer than the quickest 10% of respondents. This threshold in absolute terms was determined after reviewing the first 1,000 complete responses with correct control question answers. Respondents who answered more quickly than this absolute threshold were excluded. The established threshold was then applied to all subsequent respondents for exclusion, resulting in the exclusion of 119 responses. |
|  | *Statistical correction* | 30 | Not done. |

References

1. Eysenbach G. Improving the quality of Web surveys: the Checklist for Reporting Results of Internet E-Surveys (CHERRIES). *J Med Internet Res* 2004; 6: e34.

2. Berghofer G, Castille DM and Link B. Evaluation of Client Services (ECS): a measure of treatment satisfaction for people with chronic mental illnesses. *Community Ment Health J* 2011; 47: 399–407.

3. Franke T, Attig C and Wessel D. A Personal Resource for Technology Interaction: Development and Validation of the Affinity for Technology Interaction (ATI) Scale. *International Journal of Human–Computer Interaction* 2019; 35: 456–467.

4. Bidmon S and Terlutter R. Gender Differences in Searching for Health Information on the Internet and the Virtual Patient-Physician Relationship in Germany: Exploratory Results on How Men and Women Differ and Why. *J Med Internet Res* 2015; 17: e156.
