# Supplementary material for: Understanding citizens’ attitudes within user-centered digital health ecosystems: A sequential mixed method methodology including a web-survey
Source: Digit Health. 2024 May 20;10:20552076241255929. doi: 10.1177/20552076241255929 (PMC11418335; doi:10.1177/20552076241255929)
Supplement: sj-docx-5-dhj-10.1177_20552076241255929 - Supplemental material for Understanding citizens’ attitudes within user-centered digital health ecosystems: A sequential mixed method methodology including a web-survey [file sj-docx-5-dhj-10.1177_20552076241255929.docx]

**Original Research – Supplementary Material 5**

# Understanding citizens’ attitudes within user-centered digital health ecosystems: a sequential mixed method methodology including a web-survey

Robin Huettemann^1,5^, Benedict Sevov^1,6^, Sven Meister^2,3,7^, Leonard Fehring^1,4,8,*^

Affiliations:

1: Faculty of Health, School of Medicine, Witten/Herdecke University, Witten, Germany. *[Primary affiliation]*

2: Healthcare Informatics, Faculty of Health, School of Medicine, Witten/Herdecke University, Witten, Germany. *[Primary affiliation]*

3: Department Healthcare, Fraunhofer Institute for Software and Systems Engineering ISST, Dortmund, Germany.

4: Gastroenterology, HELIOS University Hospital Wuppertal, University Witten/Herdecke, Wuppertal, Germany.

5: ORCID: 0000-0003-3908-3029

6: ORCID: 0009-0000-2959-2394

7: ORCID: 0000-0003-0522-986X

8: ORCID: 0000-0002-3322-3724

* Corresponding author:

**Leonard Fehring**

**Address**

Witten/Herdecke University

School of Medicine

Faculty of Health

Alfred-Herrhausen-Strasse 50

58448 Witten

Germany

Email leonard.fehring@uni-wh.de

Phone +49 157 85520426

## **Supplementary Material 5.** Overview participant sample of semi-structured qualitative interviews.

All hold between 20 March and 14 April 2023.

| **Participant (P)** | **Participant personal characteristics** | **Time (in minutes)** |
| --- | --- | --- |
| P1 | Gender: Male / Age Group: ≥60 | 23 |
| P2 | Gender: Female / Age Group: <30 | 16 |
| P3 | Gender: Female / Age Group: ≥60 | 17 |
| P4 | Gender: Male / Age Group: <30 | 19 |
| P5 | Gender: Female / Age Group: ≥60 | 18 |
| P6 | Gender: Female / Age Group: ≥30-<60 | 17 |
| P7 | Gender: Male / Age Group: <30 | 21 |
| P8 | Gender: Female / Age Group: ≥30-<60 | 18 |
| P9 | Gender: Male / Age Group: ≥30-<60 | 21 |
| P10 | Gender: Female / Age Group: <30 | 17 |
| P11 | Gender: Female / Age Group: ≥60 | 20 |
| P12 | Gender: Male / Age Group: ≥60 | 16 |
| P13 | Gender: Male / Age Group: <30 | 19 |
| P14 | Gender: Male / Age Group: ≥30-<60 | 21 |
| P15 | Gender: Female / Age Group: ≥30-<60 | 17 |
